# Study of transforming growth factor alpha for the maintenance of human embryonic stem cells

**DOI:** 10.1007/s00441-012-1476-7

**Published:** 2012-08-03

**Authors:** Andy C. H. Chen, Y. L. Lee, Denise Y. C. Hou, S. W. Fong, Qian Peng, Ronald T. K. Pang, Phillip C. N. Chiu, P. C. Ho, Kai-Fai Lee, William S. B. Yeung

**Affiliations:** 1 Department of Obstetrics and Gynaecology, The University of Hong Kong, Hong Kong, China; 2 Department of Obstetrics and Gynaecology, Li Ka Shing Faculty of Medicine, The University of Hong Kong, Room 747, 21 Sassoon Road, Hong Kong, China

**Keywords:** Human embryonic stem cell, Feeder cell, Growth factor, Transforming growth factor α

## Abstract

Human embryonic stem cells (hESCs) have great potential for regenerative medicine as they have self-regenerative and pluripotent properties. Feeder cells or their conditioned medium are required for the maintenance of hESC in the undifferentiated state. Feeder cells have been postulated to produce growth factors and extracellular molecules for maintaining hESC in culture. The present study has aimed at identifying these molecules. The gene expression of supportive feeder cells, namely human foreskin fibroblast (hFF-1) and non-supportive human lung fibroblast (WI-38) was analyzed by microarray and 445 genes were found to be differentially expressed. Gene ontology analysis showed that 20.9% and 15.5% of the products of these genes belonged to the extracellular region and regulation of transcription activity, respectively. After validation of selected differentially expressed genes in both human and mouse feeder cells, transforming growth factor α (TGFα) was chosen for functional study. The results demonstrated that knockdown or protein neutralization of TGFα in hFF-1 led to increased expression of early differentiation markers and lower attachment rates of hESC. More importantly, TGFα maintained pluripotent gene expression levels, attachment rates and pluripotency by the in vitro differentiation of H9 under non-supportive conditions. TGFα treatment activated the p44/42 MAPK pathway but not the PI3K/Akt pathway. In addition, TGFα treatment increased the expression of pluripotent markers, NANOG and SSEA-3 but had no effects on the proliferation of hESCs. This study of the functional role of TGFα provides insights for the development of clinical grade hESCs for therapeutic applications.

**Electronic supplementary material:**

The online version of this article (doi:10.1007/s00441-012-1476-7) contains supplementary material, which is available to authorized users.

## Introduction

Human embryonic stem cells (hESCs) are derived from the inner cell mass of blastocysts. Their pluripotent and self-renewal properties enable their potential use in regenerative medicine (D’Amour and Gage 2000). Human ESCs were first successfully cultured on mouse embryonic fibroblast (MEF) feeder cells (Reubinoff et al. 2000; Thomson et al. 1998). To avoid possible xenogeneic contamination with the use of mouse feeder cells (Martin et al. 2005; Stacey et al. 2006), human feeder cells such as foreskin fibroblasts (Hovatta et al. 2003) and immortalized fibroblasts derived from hESCs (Xu et al. 2004) were subsequently used. However, the use of human feeder cells still has the problem of viral transmission and is labor intensive (Stacey et al. 2006). Therefore, feeder-free systems have been developed (Amit et al. 2004; Draper et al. 2004; Xu et al. 2001) but some of these systems cause chromosome instabilities and impair the cellular behavior of hESCs (Catalina et al. 2008; Maitra et al. 2005).

Feeder cells secrete growth factors for the growth and maintenance of hESCs (Amit et al. 2000; Greber et al. 2007; Xu et al. 2005). One of the growth factors produced is basic fibroblast growth factor (bFGF). Its supplementation to the culture medium is important for the self-renewal of hESCs (Xu et al. 2005). Another feeder-derived growth factor, transforming growth factor beta (TGFβ), prolongs the undifferentiated growth of hESCs when it is used together with leukemia inhibitory factor (LIF) and bFGF (Amit et al. 2004). bFGF has been reported to modulate TGFβ signaling in sustaining the pluripotency of hESCs (Greber et al. 2007).

One way to improve the feeder-free system is to identify feeder-cell-derived growth factors that are important for supporting hESCs in vitro. In this study, we have aimed at identifying these molecules by comparing the gene expression patterns of two human feeder cells, namely hFF-1 and WI-38, by using a microarray approach; the former (Hovatta et al. 2003) but not the latter cell type supports hESC culture (Richards et al. 2003). We reason that the superior abilities of hFF-1 over WI-38 are attributable to the genes that are differentially expressed in the two cell types.

## Materials and methods

### Feeder cell culture

Mouse embryonic fibroblast cell lines (STO and NIH/3T3), human foreskin fibroblast (hFF-1) and human lung fibroblasts (WI-38) were obtained from American Type Culture Collection (ATCC; Manassas, Va., USA). The feeder cells were cultured in high-glucose Dulbecco’s modified Eagle’s medium (DMEM; Gibco, Invitrogen, Calif., USA) supplemented with 15% inactivated fetal bovine serum (FBS; Gibco), 1.5 g/l sodium bicarbonate (Sigma-Aldrich, St. Louis, USA), L-glutamine (Gibco) and 1% penicillin-streptomycin (Gibco). The feeder cells were passaged every 4–7 days.

### ESC culture

hESCs, namely H9 and BG01V, were obtained from the National Stem Cell Bank (NSCB; WiCell Research Institute, USA) and American Type Culture Collection (ATCC), respectively. In the feeder system, hESCs were cultured on mitomycin-C (Invitrogen, Calif., USA)-treated human feeder layers (hFF-1 or WI-38) seeded on 0.1% gelatin (Sigma-Aldrich)-coated plates. The cells were maintained in KnockOut DMEM (KO-DMEM; Gibco) supplemented with 15% Knockout Serum Replacer (Gibco), 0.1 mM MEM non-essential amino acids (Gibco), 2 mM L-glutamine (Gibco), 0.1 mM 2-mercaptoethanol (Invitrogen) and 20 ng/ml bFGF (Invitrogen). For feeder experiments, H9 was manually dissected into fragments and cultured on human feeder layers. In the feeder-free system, hESCs were cultured on Geltrex (Invitrogen)-coated plates with StemPro-SFM (serum- and feeder-free medium; Invitrogen) supplemented with 0.1 mM 2-mercaptoethanol (Invitrogen) and 10 ng/ml bFGF (Invitrogen) with or without TGFα (R&D Systems, Minneapolis, Minn., USA) treatment. The culture medium was replaced every 48 h and the hESCs were passaged every 6 days.

### Collection of conditioned medium from human feeder cells

The conditioned medium (CM) was collected from human feeder cells (hFF-1 and WI-38) as described (McElroy and Reijo Pera 2008) with some modifications. Mitomycin-C-treated human feeder cells (hFF-1 or WI-38) were seeded at a density of 0.02×10^6^ cells/cm^2^. After being cultured in feeder medium for 24 h, the feeder cells were washed with ESC medium in the feeder system as described and further cultured in the medium at a volume of 0.3 ml/cm^2^ for 24 h. The CM were then collected and filtered through a 0.45-μm filter to remove cell debris and frozen at −20 °C for later use.

### Alkaline phosphatase activity assay

The hESC colonies were first fixed with 4% formaldehyde (freshly prepared from paraformaldehyde) for 1–2 min at room temperature. The alkaline phosphatase activity was determined by the ES Cell Characterization Kit (Chemicon, Millipore, Mass., USA) following the manufacturer’s protocol. Briefly, Fast Red Violet (0.8 g/l) was mixed with Naphthol AS-BI phosphate solution (4 mg/ml) and water in a ratio of 2:1:1 to prepare the Naphthol/Fast Red Violet solution. Fixed hESC colonies were incubated with Naphthol/Fast Red Violet Solution in the dark for 15 min. Colonies stained red were positive for alkaline phosphatase activity, whereas colorless colonies were considered negative for enzyme activity.

### Embryoid body formation

Following hESCs culture under various conditions, they were enzymatically digested with Accutase (Sigma-Aldrich). Embryoid body (EB) was formed by aggregating 25,000 cells in each well of 96-well ultra-low attachment plate (Corning, Sigma-Aldrich) by centrifugation at 300*g* for 5 min. The cells were maintained in high-glucose DMEM supplemented with 20% inactivated FBS to induce the formation of suspended EB. After 4 days of suspension culture, EBs were transferred to 0.1% gelatin-coated plates for attachment growth.

### Small interfering RNA transfection and antibody treatment

Small interfering RNAs (si-RNAs; Santa Cruz, Calif., USA) were used to down-regulate *TGFA* expression. hFF-1 was seeded at 0.025×10^6^ cells/cm^2^ and cultured for 24 h. The medium was replaced with Opti-MEM medium (Invitrogen) and the cells were transfected with 100 nM si-RNAs (scramble si-RNA and *TGFA* si-RNA; Santa Cruz) with lipofectamine 2000 (Invitrogen) for 4 h. The cells were used for ESC culture as described. Blocking of function was also performed by TGFα antibody neutralization. Mitomycin-C-inactivated hFF-1 was treated with goat serum antibody reacting with TGFα (0.8 ng/ml; R&D Systems) 1 h prior to the seeding of H9. Purified goat normal IgG (0.8 ng/ml; Zymed, Invitrogen) was used as a control.

### Gene expression profiling of human feeder cells

Mitotically inactivated hFF-1 and WI-38 were cultured on 10-cm culture plates for 1 day before trypsinization with 0.05% trypsin-EDTA (Invitrogen). Total RNA was extracted from hFF-1 and WI-38 (*n*=3) samples from various passages by the RNeasy Mini Kit (Qiagen, Crawley, Sussex, UK). The qualities of the extracted RNA were examined by an Agilent 2100 bioanalyzer (Waldboronn, Germany) and 1 μg total RNA from each sample was used for gene profiling by the GeneChip system (Human Genome U133 Plus 2.0 microarray, Affymetrix, Santa Clara, Calif., USA), which was composed of more than 54,000 probes targeted at over 47,000 human transcripts and variants.

### Reverse transcription and real-time quantitative polymerase chain reaction

Total RNAs from hFF-1, WI-38, STO and NIH/3T3 were extracted by the mirVana PARIS Kit (Ambion, Austin, USA) and reverse-transcribed by TaqMan Reverse Transcription Reagents (Applied Biosystems, Foster City, Calif., USA). Relative quantification of mRNA was performed by real-time quantitative PCR (qPCR) in a 7500 Real-Time PCR System (Applied Biosystems) and normalized with endogenous 18S ribosomal RNA by using the 2^−∆∆CT^ method. The data were analyzed by the manufacturer’s software (Applied Biosystems).

### Western blot analysis

Cells were lysed with the cell disruption buffer of the mirVana PARIS Kit (Ambion) supplemented with protease and phosphatase inhibitors (Calbiochem, Darmstadt, Germany). Equal amounts of protein were heat-inactivated, resolved in 10% or 12% polyacrylamide gels and transferred to polyvinylidene fluoride membrane for Western blot analysis with antibodies against cell-signaling molecules (Cell Signaling, Mass., USA): phosphotyrosine, phospho-p44/42 mitogen-activated protein kinase (p44/42 MAPK), phospho-AKT (Ser473), phospho-AKT (Thr308), AKT, NANOG (R&D, Minn., USA) and proliferating cell nuclear antigen (PCNA; Dako, Glostrup, Denmark), or β-ACTIN (Sigma-Aldrich) for normalization.

### Flow cytometric analysis

H9 was trypsinized and resuspended in phosphate-buffered saline (PBS) at 10^6^ cells/ml. For cell-surface staining of stage-specific embryonic antigen-3 (SSEA-3; BD Biosciences, San Jose, Calif., USA), H9 were fixed in 4% formaldehyde (freshly prepared from paraformaldehyde), followed by incubation with phycoerythrin-conjugated anti-SSEA-3 antibody. For nuclear staining of the proliferation marker, H9 was first incubated with 10 μM BrdU for 1 h prior to fixation in methanol. The cells were then incubated successively with sheep serum antibodies reacting with BrdU (Abcam, Cambridge, Mass., USA) and fluorescein-isothiocyanate-conjugated rabbit serum antibodies reacting with sheep-IgG (Zymed, Invitrogen). The percentage of fluorescently labeled cells was analyzed by flow cytometry (FACSCanto II, BD Biosciences) by using the software WinMDI 2.9 (The Scripps Research Institute, Calif., USA).

### Fluorescent immunocytochemical staining of pluripotent and differentiation markers

The hESC colonies or EB were washed with PBS and fixed with 4% formaldehyde (freshly prepared from paraformaldeyhde), followed by permeabilization with 0.1% Triton. Normal goat or rabbit serum was used for blocking prior to incubation with the appropriate primary antibody diluted in blocking solution at 4°C overnight. Subsequently, the cells were incubated with the corresponding secondary antibody for 1 h at room temperature in the dark. The nuclei of the cells were stained with propidium iodide or 4′-6-diamidino-2-phenylindole. The fluorescent images were observed under an inverted fluorescence microscope (Nikon, Japan) equipped with a digital camera (Sensys, Photometrics, Tucson, USA) or a confocal microscope (LSM 700, Carl Zeiss, Oberkochen, Germany). The antibodies used were against SSEA-4, TRA-1-60, TRA-1-81 (Chemion, Millipore, Mass., USA), Nanog (R&D), OCT4 (Santa Cruz), or KRT-18 (Abcam).

### Statistical analysis

Data were analyzed and plotted by using SigmaPlot software (Aspire Software International, Leesburg, Va., USA). One-way analysis of variance (ANOVA) followed by the Student-Newman Keuls method/Rank Sum Test were used to compare the results. A *P* value <0.05 was considered to be statistically significant.

## Results

### hFF-1 but not WI-38 supported the culture of hESC

The two hESC lines used in this study, H9 and BG01V, were first characterized. Positive alkaline phosphatase activities were found in both H9 and BG01V (Suppl. Fig. 1a). Immunocytochemistry indicated that the colonies stained positively for pluripotent markers (NANOG, OCT4, TRA-1-60, TRA-1-81, SSEA-4) and gap junction molecules (CX-43), whereas the expression of the early differentiation marker KRT18 (Cauffman et al. 2009) was minimal (Suppl. Fig. 1b). Upon in-vitro differentiation of H9 and BG01V by the formation of EB, positive immunoreactivities of the three germ layer markers (mesoderm: muscle-actin; ectoderm: tubulin-βIII; endoderm: α-fetoprotein) were present in the cells, indicating that they were pluripotent under our culture conditions (Suppl. Fig. 1c).

**Fig. 1:**
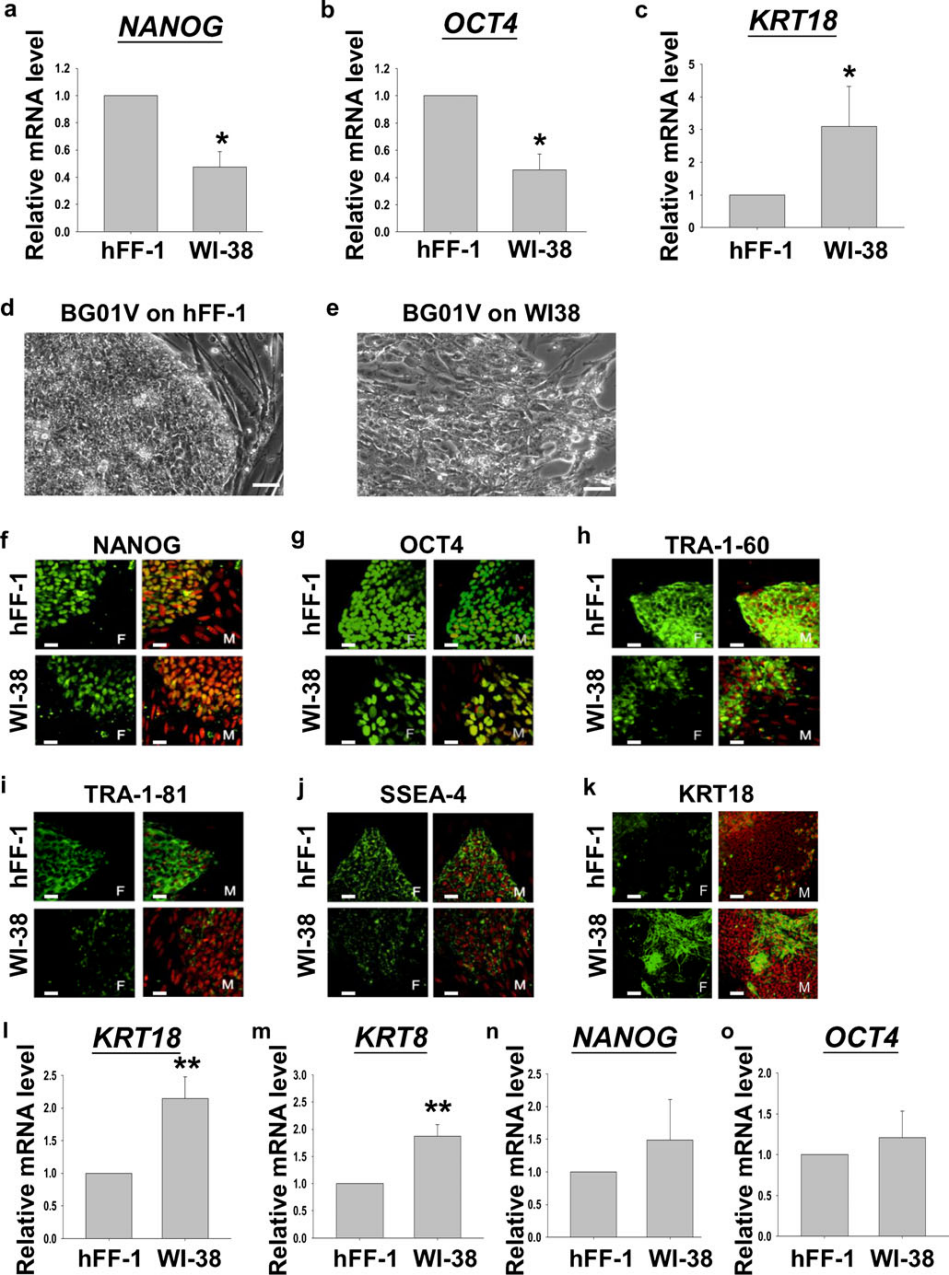
**a–c** Relative mRNA levels of *NANOG*, *OCT4* and *KRT18* of H9 cultured on hFF-1 or WI-38 feeder layers (*n*=4). **d**, **e** Representative images of BG01V cultured on hFF-1 or WI-38 for 6 days. *Bars* 5 μm. **f–k** Immunofluorescent staining of pluripotent markers (NANOG, OCT4, TRA-1-60, TRA-1-81, SSEA4) and early differentiation marker (KRT18) in BG01V cultured on hFF-1 and WI-38 (*F* fluorescence, *M* merged images of fluorescence and propidium iodide). *Bars* 5 μm. **l–o** Relative mRNA levels of the early differentiation markers (*KRT8*, *n*=7; *KRT18*, *n*=9) and pluripotent markers (*NANOG*, *n*=5; *OCT4*, *n*=9) in BG01V cultured on hFF-1 and WI-38. *P<0.05, **P<0.01

Previously, hFF-1 (Hovatta et al. 2003) but not WI-38 (Dravid et al. 2005) was reported to support the undifferentiated growth of H9 (hESC used for the functional study in the present study). Our data also demonstrated that H9 grown on hFF-1 had higher pluripotent marker expressions (*NANOG* and *OCT4*) and lower differentiation marker expression (*KRT18*) than that on WI-38 (Fig. 1a, *P*<0.05, Rank Sum Test). In this study, we used another hESC line, namely BG01V, to confirm further the supportive roles of hFF-1 on hESC growth. The morphologies of BG01V grown on inactivated hFF-1 or WI-38 for 6 days were compared. On day 6 of culture, the boundaries of BG01V colonies cultured on WI-38 became non-distinctive and differentiating colonies were noted. On the contrary, the borders of the colonies growing on hFF-1 remained distinctive with minimal differentiation (Fig. 1b). The colonies were then graded into three classes based on the extent of differentiation (shape, thickness and fragility) according to previous studies (Heng et al. 2006; Richards et al. 2004; Grade A: >90% undifferentiated; Grade B: 50–90% undifferentiated; Grade C: <50% undifferentiated). The grading was performed by two blinded observers: significantly more colonies were graded as “A” on hFF-1 (51.7%) when compared with WI-38 (7.78%), and the number of colonies graded as “C” on hFF-1 (5%) was significantly lower than that on WI-38 (33.3%). Immunofluorescent staining of BG01V cultured on hFF-1 and WI-38 showed overall diminished intensities of pluripotent markers (NANOG, OCT4, TRA-1-60, TRA-1-81 and SSEA-4) but an enhanced intensity of the early differentiation marker, KRT18, in BG01V grown on WI-38 (Fig. 1c). We then compared the mRNA expressions of the pluripotent (*NANOG*, *OCT4*) and early differentiation (*KRT8*, *KRT18*) markers in these cells; we found that *KRT8* and *KRT18* were significantly higher in BG01V grown on WI-38 when compared with those growing on hFF-1 (Fig. 1d; *P*<0.01, Rank Sum Test), although no significant difference was found in the pluripotent markers. The data further confirmed that hFF-1 supported the undifferentiated growth of hESCs.

### Differential gene expression in hFF-1 and WI-38

**Fig. 2:**
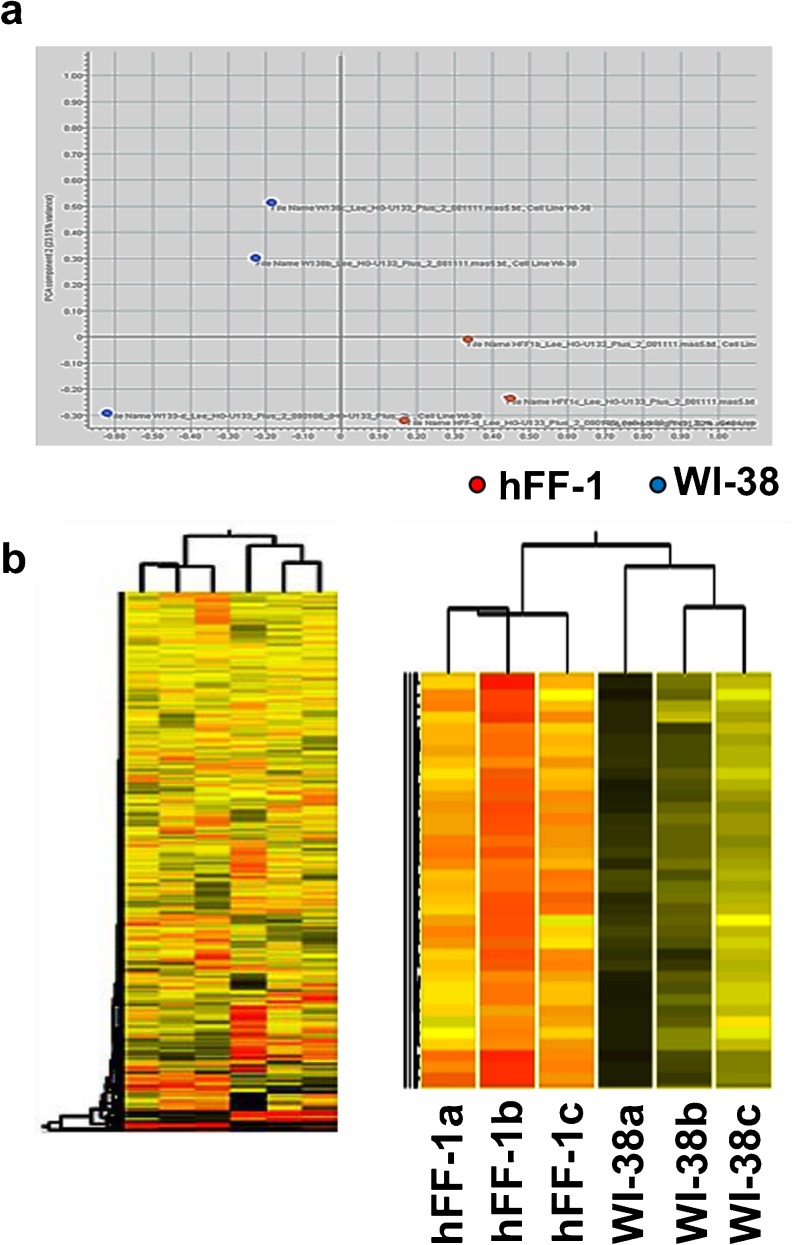
**a** Principal component analysis diagram of the microarray data to examine the differences in the overall gene expression profiles of hFF-1 (*n*=3, *red dots*) and WI-38 (*n*=3, *blue dots*). **b** Tree diagram of unsupervised cluster analysis of the microarray data on three independent samples of hFF-1 (*a–c*) and WI-38 (*a–c*). *Red regions* Highly expressed genes. *Green/brown regions* Genes expressed at a lower level

To improve our understanding of the way that feeder cells support the pluripotency of hESCs, we used microarray analysis to compare the gene expression pattern between three independent samples of the supportive hFF-1 and the non-supportive WI-38. Principal component analysis indicated that the hFF-1 samples were separated from the WI-38 samples (Fig. 2a). In the unsupervised cluster analysis, clustering of the hFF-1 samples from the WI-38 samples was found (Fig. 2b). A total of 445 differentially up-regulated genes (fold change >2; *P*<0.05) in the hFF-1 cells was identified (Suppl. Table S1).

**Fig. 3:**
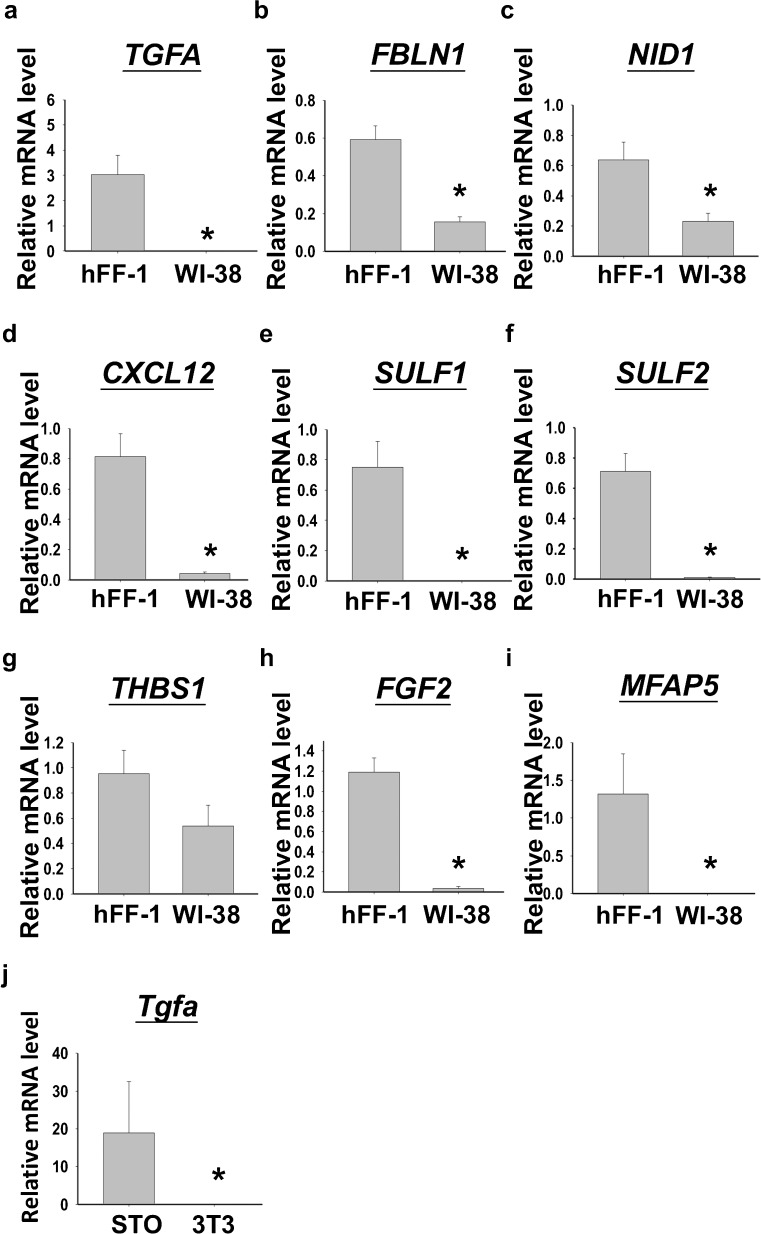
**a–i** Quantitative polymerase chain reaction analysis for the validation of selected gene expression between hFF-1 (*n*=10) and WI-38 (*n*=6). **j** Relative mRNA levels of transforming growth factor alpha (*Tgfa*) in mouse feeder cells, namely STO and NIH/3T3 (*n*=5). *P<0.05

Among the genes differentially up-regulated (total 445) in hFF-1 cells, gene ontology analysis showed that 93 (20.9%) belonged to the extracellular region and 69 (15.5%) were involved in the regulation of transcription activity (Suppl. Table S2). These genes were potential beneficial factors for the maintenance of hESCs. In this study, nine genes were selected for validation by qPCR. They were selected based on the finding that they were for extracellular matrix (ECM) proteins, interacting partners of ECM proteins, molecules that were related to cell adhesion, or molecules interacting with pathways reported to be important in the regulation of hESC pluripotency. Except for thrombospondin-1 (*THBS1*), the expression of the other eight genes including *TGFA*, fibulin-1 (*FBLN1*), nidogen-1 (*NID1*), chemokine (C-X-C motif) ligand-12 (*CXCL12*), sulfatase-1 and −2 (*SULF1* and *SULF2*), *FGF2* and microfibrillar associated protein-5 (*MFAP5*) were significantly higher in the hFF-1 cells when compared with those in the WI-38 cells (Fig. 3a, *P*<0.05, *t*-test).

*TGFA* was chosen for further validation because of its reported action on mouse ESC (mESCs; Heo et al. 2008). We first compared *tgfa* expression in two mouse fibroblast cell lines, namely STO and NIH/3T3; the former was supportive (Park et al. 2003, 2004), whereas the latter was non-supportive (Wang et al. 2005) to hESC culture. The results showed that the *tgfa* mRNA level was significantly higher in the supportive STO cells when compared with the non-supportive NIH/3T3 cells (Fig. 3b, *P*<0.05, *t*-test). As both human and mouse supportive feeder cells expressed higher levels of *TGFA*, we postulated that it was involved in maintaining the undifferentiated growth of hESC.

### Knock-down or protein neutralization of TGFα in hFF-1 reduced H9 attachment and pluripotent marker gene expressions

**Fig. 4:**
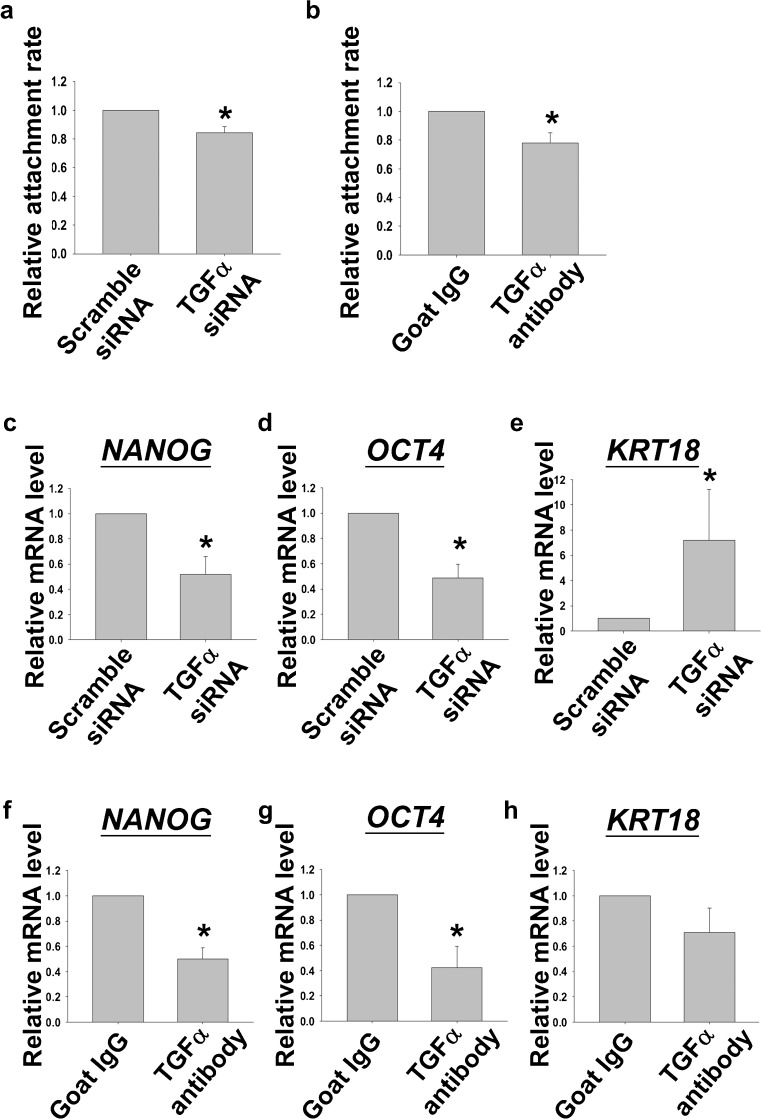
**a**, **b** Relative attachment rates of H9 after seeding on hFF-1 transfected with scramble small interfering RNA (si-RNA) or *TGFA* si-RNA or on hFF-1 pre-treated with goat IgG or antibody against TGFα (*n*=4). **c–e** Relative mRNA levels of *NANOG*, *OCT4* and *KRT18* in H9 cultured on hFF-1 transfected with scramble si-RNA or *TGFA* si-RNA (*n*=4). **f–h** Relative mRNA levels of *NANOG*, *OCT4* and *KRT18* in H9 cultured on hFF-1 pre-treated with goat IgG or antibody against TGFα (*n*=4). *P<0.05)

In the present study, we sought to study the biological role of TGFα on the maintenance of hESC. Cell attachment is important for the propagation and maintenance of hESC culture. In this section, the effects of the knockdown of *TGFA* expression by si-RNA or protein neutralization by antibody against TGFα on H9 were examined. The attachment of H9 was investigated by comparing the number of H9 fragments attached to feeder layers with or without treatments at 24 h after seeding. The attachment rates of H9 fragments cultured on the hFF-1 feeder layer pre-treated with *TGFA* si-RNA (100 nM) or TGFα antibody (0.8 ng/ml) were found to be significantly lower when compared with those of controls (Fig. 4a, *P*<0.05, Rank Sum Test).

H9 cultured on an hFF-1 feeder layer pre-treated with *TGFA* si-RNA (100 nM) or TGFα antibody (0.8 ng/ml) were found significantly to reduce the expression levels of *NANOG* and *OCT4* when compared to their respective controls. On the other hand, the expression level of *KRT18* was significantly elevated in the *TGFA* si-RNA treatment group (Fig. 4b, c, *P*<0.05, Rank Sum Test) but not after antibody treatment.

### TGFα rescued the maintenance of hESC culture and its pluripotency under non-supportive conditions

**Fig. 5:**
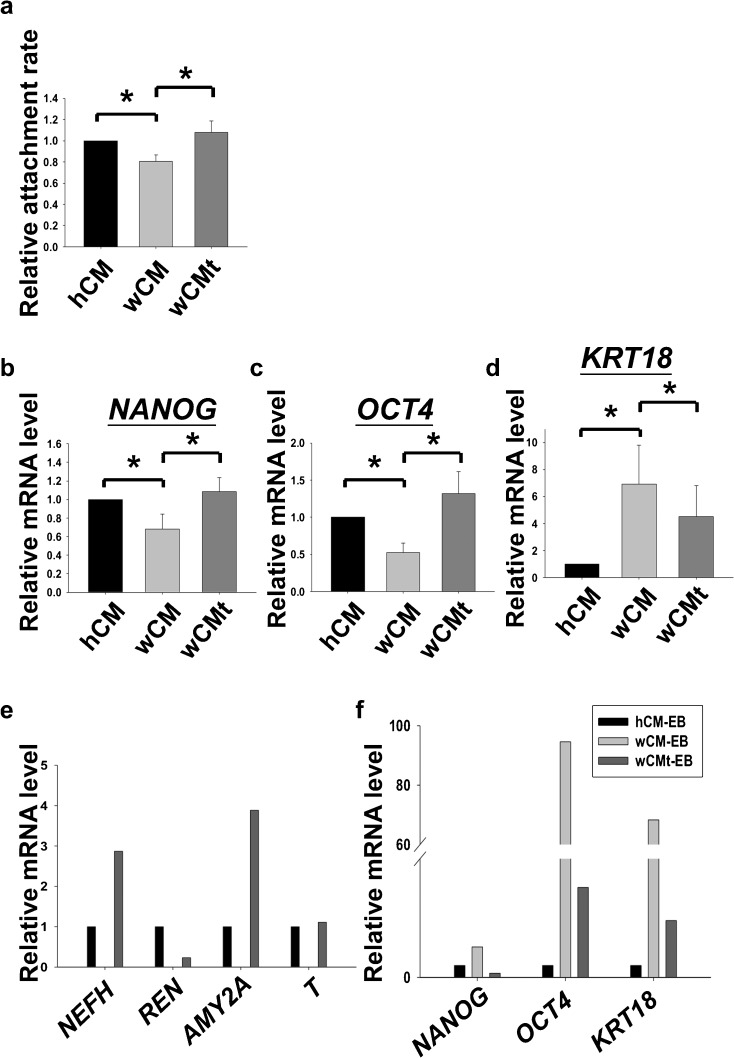
**a** Relative attachment rates of H9 after cultured in hFF-1 conditioned medium (hCM), WI-38 CM (wCM), or WI-38 CM supplemented with TGFα (wCMt), *n*=5. **b–d** Relative mRNA levels of *NANOG*, *OCT4* and *KRT18* of H9 cultured in hCM, wCM, or wCMt (*n*=4). **e**, **f** Relative mRNA levels of three germ layer markers (mesoderm: *REN* and *T*; ectoderm: *NEFH*; endoderm: *AMY2A*), pluripotent markers (*NANOG*, *OCT4*) and early differentiation marker (*KRT18*) in embryoid body formed from differentiation of H9 cultured in hCM (*hCM-EB*), wCM (*wCM-EB*), or wCMt (*wCMt-EB*)

Our results demonstrated that TGFα knockdown or protein neutralization in hFF-1 suppressed the attachment rates and pluripotent marker gene expression in H9. Next, we sought to investigate the effects of recombinant TGFα supplementation on the growth of H9. H9 was grown in WI-38 CM with (wCMt) or without (wCM) the supplementation of recombinant TGFα (100 ng/ml) and compared with hFF-1 CM (hCM). As expected, the attachment rate of H9 fragments was significantly higher in hCM when compared with wCM. The expression levels of pluripotent markers *NANOG* and *OCT4* were significantly lower, whereas the expression of the differentiation gene *KRT18* was significantly higher in H9 cultured in wCM. The supplementation of TGFα to wCM (wCMt) was found to restore the attachment rate and the expression levels of *NANOG* and *OCT4* in H9 to that in hCM. The expression of *KRT18* in H9 cultured in wCMt was also shown to be significantly down-regulated (Fig. 5a, b, *P*<0.05, Rank Sum Test).

To investigate whether TGFα supported the pluripotency of hESC, H9 cultured in hCM, wCM, or wCMt were subjected to in vitro differentiation by the formation of EB. We found that suspended EB with similar sizes were formed on Day 4 among these three groups (data not shown). The EBs were subjected to attachment growth until Day 8. All the EB attached successfully onto the culture plate. However, EBs of wCM (wCM-EB) were found to have fewer outgrowths than those of hCM (hCM-EB; Suppl. Fig. 2). Interestingly, EBs of the wCMt groups (wCMt-EB) spread out on the culture plate and retained a similar morphology to that of hCM-EB (Suppl. Fig. 2). The mRNA expression of the three germ layer markers in EB (mesoderm: *REN* and *T*; ectoderm: *NEFH*; endoderm: *AMY2A*) were analyzed. Surprisingly, all three germ layer markers were absent in wCM-EB, whereas the expression levels of pluripotent markers (*NANOG* and *OCT4*) and early-differentiation marker (*KRT18*) were found to be much higher in wCM-EB when compared with those in hCM-EB. On the contrary, all the germ layer markers were detected in wCMt-EB. In addition, the expression levels of the pluripotent markers and early differentiation marker in wCMt-EB were suppressed when compared with those in wCM-EB (Fig. 5c). The findings demonstrated that TGFα helped in the maintenance of hESC and retained its pluripotency in non-conditioned medium.

### TGFα activated the p44/42 MAPK but not the PI3K/Akt pathway of hESCs

**Fig. 6:**
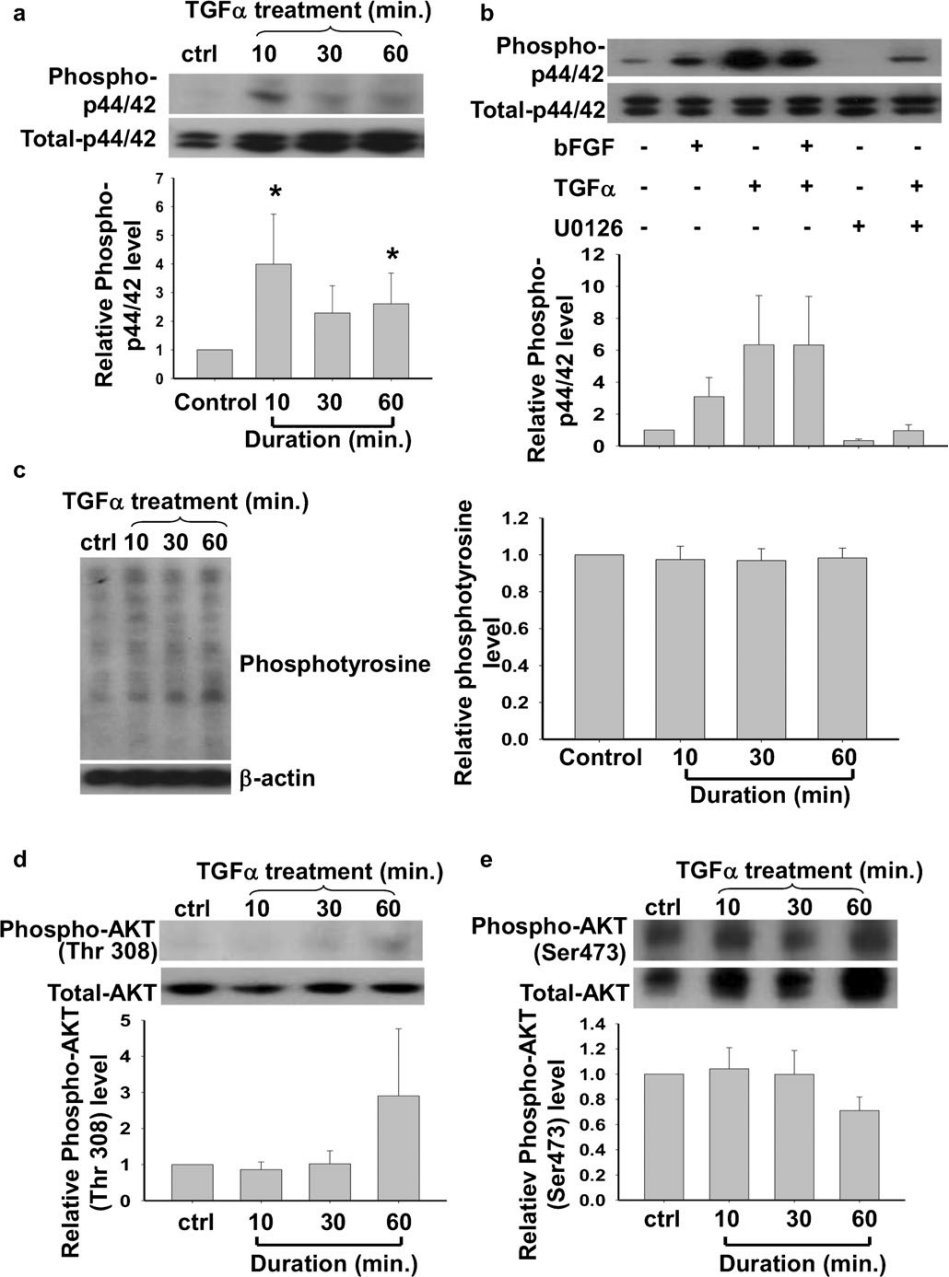
**a**, **b** Effects of TGFα on the relative expression levels of phosphorylated p44/42 in H9 after treated with (**a**) TGFA for 10, 30, or 60 mins (*n*=5; *ctrl* control) and (**b**) basic fibroblast growth factor (*bFGF*), TGFα and/or U0126 (*n*=5). **c** Relative level of phosphotyrosine in H9 after TGFα treatment (*n*=7). **d**, **e** Relative level of phosphorylated Akt at Thr308 and Ser473 in H9 after TGFA treatment (*n*=6). Statistical analysis was performed by one way analysis of variance. *P<0.05

After we had confirmed the biological role of TGFα in the maintenance of pluripotency of hESC, we studied the signaling pathways induced by TGFα in hESC. H9 cells were cultured in a feeder-free system without bFGF for 24 h before treatment with 100 ng/ml recombinant human TGFα for 0, 10, 30, or 60 min. TGFα activated the MAPK pathway, as demonstrated, by a significantly higher expression of phosphorylated p44/42 after the 10- and 60-min treatments (Fig. 6a, *P*<0.05). The stimulatory action of TGFα on phosphorylated p44/42 was suppressed by a mitogen-activated protein kinase kinase (MEK) inhibitor, U0126. No additive effect on the stimulation of the MAPK pathway was found when H9 was treated with both bFGF and TGFα (Fig. 6b). However, TGFα treatment did not activate the phosphatidylinositol-3-kinase (PI3K)/AKT pathway in hESCs as the levels of phosphotyrosine (Fig. 6c) and phosphorylated AKT (Fig. 6d, e) were not affected by TGFα treatments.

### TGFα increased the expression of pluripotent markers NANOG and SSEA-3 in hESCs

**Fig. 7:**
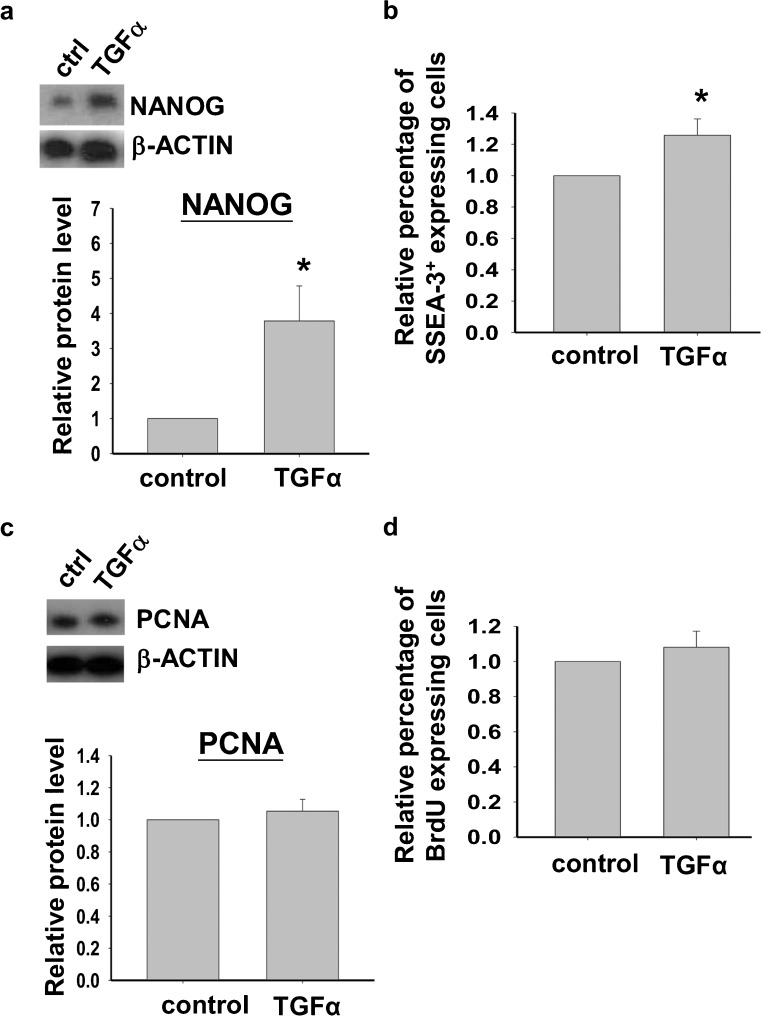
**a**, **b** Effect of TGFα on the expression of the pluripotent marker, NANOG, by Western blotting analysis (*n*=5) and the percentage of cells expressing the pluripotent marker, SSEA-3, by flow cytometry analysis (*n*=7), respectively. **c**, **d** Effect of TGFα on the relative expression levels of proliferation markers, namely proliferating cell nuclear antigen (*PCNA*) protein by Western blotting analysis (*n*=4) and BrdU incorporation by flow cytometric analysis (*n*=5). *P<0.05)

The biological role of TGFα on the maintenance of hESC was then studied. First, we investigated the functional role of TGFα on the maintenance of undifferentiated growth of hESC. After a 24-h treatment, TGFα (100 ng/ml) significantly increased the protein expression of the pluripotent marker, NANOG, when compared with the control (Fig. 7a, *P*<0.05). Flow cytometric analysis also showed that TGFα increased the percentage of cells expressing the surface pluripotent marker, SSEA-3 (Fig. 7b).

### TGFα did not affect proliferation of hESCs

The effect of TGFα on the proliferation of H9 was then studied. However, our findings demonstrated that TGFα had no effect on the protein expression level of a cell proliferation marker, PCNA, as shown by using Western blotting analysis (Fig. 7c). In addition, flow cytometric analysis revealed that treatment of TGFα had no effect on BrdU incorporation (Fig. 7d). The results implied that H9 cell proliferation was not affected by TGFα treatment.

## Discussion

Human ESCs are commonly derived and propagated on inactivated human or mouse feeder cells by using commercially available knockout cell culture medium containing bFGF. Current researches into hESC culture have concentrated on the development of defined culture conditions without the use of feeder cells (Li et al. 2005; Ludwig et al. 2006). Feeder cells have been suggested to provide ECM molecules and soluble factors to maintain the pluripotency of hESCs in culture (Assou et al. 2007). The combined use of noggin, an MEF-expressing factor and bFGF can maintain the pluripotent growth of hESCs under feeder-free conditions (Wang et al. 2005). However, hESC culture with the defined medium still requires xenogenic matrigel that might contain undefined contaminants (Curry 2009). Further searches for the optimal hESC culture condition are needed.

The gene expression patterns of growth factors between supportive human and mouse feeder cells are different. Human but not mouse, feeder cells secrete bFGF (Eiselleova et al. 2008). On the other hand, the latter secrete more activin A than the former (Eiselleova et al. 2008). Thus, mouse and human feeder cells might support hESC growth via different pathways. By using another hESC line, BG01V, we have further confirmed the non-supportive role of WI-38 (Dravid et al. 2005; Richards et al. 2003) on maintaining the undifferentiated growth of hESC as demonstrated by higher early differentiation marker expression in these cells. The lack of effect on the expression of *NANOG* and *OCT4* in BG01V cultured on WI-38 might be attributable to the abnormal karyotyping, which confers higher resistance to differentiation (Zeng et al. 2004). Based on this rationale, H9 with a normal karyotype has been used for subsequent functional assays in this study. To improve our understanding of the way that feeder cells support pluripotency of hESCs, we have used a genomic approach to compare the gene expression pattern between the supportive hFF-1 and the non-supportive WI-38 feeder cells. Previous studies have used the transcriptomic approach (Kueh et al. 2006; Villa-Diaz et al. 2009) and proteomic approach (Chin et al. 2007; Prowse et al. 2007, 2005) to compare the gene expression patterns between supportive and non-supportive feeder cells. In agreement with a similar study involving the use of the serial analysis of gene expression (SAGE; Kueh et al. 2006) to compare the gene expression patterns between supportive human fetal skin fibroblasts and non-supportive human embryonic lung fibroblasts, several genes including ECM components such as fibronectin, sulfatase-1 and collagen α-1 and growth factors such as bFGF and TGFα have also been found to be highly expressed in supportive hFF-1 in the present study. Coincidentally, high levels of fibronectin and collagen α-1 have been detected in hFF-conditioned media (Prowse et al. 2007).

Gene ontology analysis of the transcriptomic data suggests that many of the genes differentially up-regulated in the supportive hFF-1 cells belong to the extracellular region or are involved in the regulation of transcription. Therefore, genes belonging to these groups have been selected for validation. Among the validated genes, bFGF is well-known for hESC self-renewal (Greber et al. 2007). Nidogen-1 is an ECM protein that promotes hESC assembly (Evseenko et al. 2009). CXCL12 enhances the survival of mESCs (Guo et al. 2005). TGFα is involved in cell proliferation and the maintenance of pluripotency in mESCs (Heo et al. 2008). However, the role of TGFα on hESC culture was previously unknown. Therefore, this study has focused on the functions of TGFα on hESCs.

The negative effects of TGFα knockdown and antibody neutralization on H9 suggest a critical role of TGFα in maintaining the undifferentiated growth of hESC. Most importantly, the supplementation of TGFα to non-supportive WI-38 CM partially rescues the pluripotent state of H9, as shown by the increased levels of pluripotent marker expression and the presence of marker expression for the three germ layers upon in vitro differentiation. Added together, our findings suggest that TGFα is involved in maintaining the self-renewal and pluripotency of hESCs.

TGFα is a member of the epidermal growth factor (EGF) family. It binds to the epidermal growth factor receptor (EGFR) and triggers multiple downstream signaling pathways that are important in cell proliferation and survival (Ferrer et al. 1996; Jorissen et al. 2003; Prenzel et al. 2000; Santa-Olalla and Covarrubias 1995; Tang et al. 1997). TGFα has been reported to be able to activate the MAPK signaling pathway in cancer cells (Sawhney et al. 2004; Zheng et al. 2008). Interestingly, TGFα also activates the MAPK, PI3-K/AKT and Notch signaling pathways in mESCs (Heo et al. 2008). However, the effect of TGFα on hESC growth is unclear.

Our data demonstrate the activation of the p44/42 MAPK pathway by TGFα. The effect is initiated rapidly within 10 min after TGFα treatment. The observation is in agreement with the finding that TGFα stimulates the MAPK cascade in mESCs (Heo et al. 2008). Since bFGF has been reported to stimulate the MAPK pathway through FGF receptors (Eiselleova et al. 2009), we have tested whether bFGF has an additive effect with TGFα on MAPK pathway activation. However, no additive effect has been found when H9 is treated with both bFGF and TGFα, suggesting that the treatment with TGFα might have activated the highest level of p44/42 MAPKs.

In the present study, the knockdown of *TGFA* expression or neutralization of TGFα leads to lower attachment rates of H9 fragments. Previous studies have demonstrated that TGFα activates the MAPK pathway leading to the increased expression of integrin α2 for cellular adhesion in colon cancer cells (Sawhney et al. 2004). Interestingly, the exogenous FGF-2-activated MAPK signaling pathway in hESC is also involved in increasing hESC attachment and cloning efficiency (Eiselleova et al. 2009). Our data on TGFα-induced MAPK signaling and cell attachment suggest that TGFα has a similar function to FGF-2 in maintaining the undifferentiated growth of hESCs. In addition, under feeder-free culture conditions, TGFα treatment increases the expression of pluripotent markers, NANOG and SSEA-3. NANOG is involved in maintaining the self-renewal of ES cells (Chambers and Smith 2004). The overexpression of NANOG might maintain the self-renewal of mESCs in the absence of LIF (Mitsui et al. 2003). Our findings suggested that TGFα is involved in maintaining the self-renewal of hESCs. The substitution of bFGF with TGFα for the long-term culture of undifferentiated hESC needs further investigation.

Our data have shown that the actions of TGFα on hESCs are different from their actions on mESC. First, TGFα does not activate the PI3-K/AKT pathway in hESCs. Second, it has no effect on hESC proliferation. TGFα stimulates the growth of glioma cells through the phosphorylation of ERK1/2 (Zheng et al. 2008). TGFα can also reduce the apoptotic rate induced by tumor necrosis factor-α (TNFα) in human keratinocytes (Reinartz et al. 1996). In mESs, TGFα increases the cell proliferation rate and promotes DNA synthesis through the stimulation of the MAPK, PI3-K/Akt and Notch signaling pathways (Heo et al. 2008). In addition, TGFα inhibits apoptosis in mESCs (Heo et al. 2008), whereas increased apoptosis has been found in TGFα-deficient mouse blastocysts (Brison and Schultz 1998). However, our data demonstrate no proliferative effect of TGFα on the growth of hESC. This discrepancy of TGFα effects on mESC and hESC might be explained in part by the finding that the activation of the MAPK pathway maintains the pluripotency but does not increase the proliferation rate of hESCs (Li et al. 2007).

In conclusion, TGFα is expressed at a higher level in the supportive human and mouse feeder cells, hFF-1 and STO. Blockage of TGFα function leads to lower attachment rates and pluripotent marker gene expression. On the other hand, the supplementation of TGFα in a non-supportive conditioned medium can maintain the pluripotent gene expression, attachment rates and pluripotency of hESC. TGFα has been found to activate the p44/42 MAPKs pathway and to enhance the expression of pluripotent markers but not the proliferation of H9. Further investigations into the roles of other differentially expressed genes found in this study are undergoing.

## Electronic supplementary material

**Suppl. Fig. 1** **a** Alkaline phosphatase staining of H9 and BG01V cultured on hFF-1. *Bar* 5 μm. **b** Immunofluorescent staining of pluripotent markers (NANOG, OCT4, TRA-1-60, TRA-1-81, SSEA4) and early differentiation marker (KRT18) in H9 and BG01V cultured on hFF-1 (*F* fluorescence, *M* merged). *Bar* 5 μm. **c** Immunofluorescent staining of germ layer markers (muscle actin, tubulin β-III, and α-fetoprotein) of embryoid body derived from H9 and BG01V. *Bar* 5 μm.

**Suppl. Fig. 2** Representative images of embryoid body derived from H9 that have been cultured (*CM* conditioned medium) under various culture conditions (hFF-1 CM, WI-38 CM, and WI-38 CM with the supplementation of TGFα). *Bar* 5 μm.

ESM 1 (PDF 698 kb)

ESM 2 (DOC 610 kb)

## Abbreviations

bFGF: Basic fibroblast growth factor
EB: Embryoid body
ECM: Extracellular matrix
hESC: Human embryonic stem cell
hFF-1: Human foreskin fibroblast
LIF: Leukemia inhibitory factor
MAPK: Mitogen-activated protein kinase
MEF: Mouse embryonic fibroblast
mES: Mouse embryonic stem cells
PCNA: Proliferating cell nuclear antigen
PI3-K: Phosphoinositide 3-kinase
TGFA: Transforming growth factor α
TGFΒ: Transforming growth factor β
WI-38: Human lung fibroblast

## References

[CR1] Amit M, Carpenter MK, Inokuma MS, Chiu CP, Harris CP, Waknitz MA, Itskovitz-Eldor J, Thomson JA (2000). Clonally derived human embryonic stem cell lines maintain pluripotency and proliferative potential for prolonged periods of culture. Dev Biol.

[CR2] Amit M, Shariki C, Margulets V, Itskovitz-Eldor J (2004). Feeder layer- and serum-free culture of human embryonic stem cells. Biol Reprod.

[CR3] Assou S, Le Carrour T, Tondeur S, Strom S, Gabelle A, Marty S, Nadal L, Pantesco V, Reme T, Hugnot JP (2007). A meta-analysis of human embryonic stem cells transcriptome integrated into a web-based expression atlas. Stem Cells.

[CR4] Brison DR, Schultz RM (1998). Increased incidence of apoptosis in transforming growth factor alpha-deficient mouse blastocysts. Biol Reprod.

[CR5] Catalina P, Montes R, Ligero G, Sanchez L, de la Cueva T, Bueno C, Leone PE, Menendez P (2008). Human ESCs predisposition to karyotypic instability: is a matter of culture adaptation or differential vulnerability among hESC lines due to inherent properties?. Mol Cancer.

[CR6] Cauffman G, De Rycke M, Sermon K, Liebaers I, Van de Velde H (2009). Markers that define stemness in ESC are unable to identify the totipotent cells in human preimplantation embryos. Hum Reprod.

[CR7] Chambers I, Smith A (2004). Self-renewal of teratocarcinoma and embryonic stem cells. Oncogene.

[CR8] Chin AC, Fong WJ, Goh LT, Philp R, Oh SK, Choo AB (2007). Identification of proteins from feeder conditioned medium that support human embryonic stem cells. J Biotechnol.

[CR9] Curry S (2009). Lessons from the crystallographic analysis of small molecule binding to human serum albumin. Drug Metab Pharmacokinet.

[CR10] D’Amour K, Gage FH (2000). New tools for human developmental biology. Nat Biotechnol.

[CR11] Draper JS, Moore HD, Ruban LN, Gokhale PJ, Andrews PW (2004). Culture and characterization of human embryonic stem cells. Stem Cells Dev.

[CR12] Dravid G, Ye Z, Hammond H, Chen G, Pyle A, Donovan P, Yu X, Cheng L (2005). Defining the role of Wnt/beta-catenin signaling in the survival, proliferation, and self-renewal of human embryonic stem cells. Stem Cells.

[CR13] Eiselleova L, Peterkova I, Neradil J, Slaninova I, Hampl A, Dvorak P (2008). Comparative study of mouse and human feeder cells for human embryonic stem cells. Int J Dev Biol.

[CR14] Eiselleova L, Matulka K, Kriz V, Kunova M, Schmidtova Z, Neradil J, Tichy B, Dvorakova D, Pospisilova S, Hampl A (2009). A complex role for FGF-2 in self-renewal, survival, and adhesion of human embryonic stem cells. Stem Cells.

[CR15] Evseenko D, Schenke-Layland K, Dravid G, Zhu Y, Hao QL, Scholes J, Wang XC, Maclellan WR, Crooks GM (2009). Identification of the critical extracellular matrix proteins that promote human embryonic stem cell assembly. Stem Cells Dev.

[CR16] Ferrer I, Alcantara S, Ballabriga J, Olive M, Blanco R, Rivera R, Carmona M, Berruezo M, Pitarch S, Planas AM (1996). Transforming growth factor-alpha (TGF-alpha) and epidermal growth factor-receptor (EGF-R) immunoreactivity in normal and pathologic brain. Prog Neurobiol.

[CR17] Greber B, Lehrach H, Adjaye J (2007). Fibroblast growth factor 2 modulates transforming growth factor beta signaling in mouse embryonic fibroblasts and human ESCs (hESCs) to support hESC self-renewal. Stem Cells.

[CR18] Guo Y, Hangoc G, Bian H, Pelus LM, Broxmeyer HE (2005). SDF-1/CXCL12 enhances survival and chemotaxis of murine embryonic stem cells and production of primitive and definitive hematopoietic progenitor cells. Stem Cells.

[CR19] Heng BC, Vinoth KJ, Liu H, Hande MP, Cao T (2006). Low temperature tolerance of human embryonic stem cells. Int J Med Sci.

[CR20] Heo JS, Lee SH, Han HJ (2008). Regulation of DNA synthesis in mouse embryonic stem cells by transforming growth factor-alpha: involvement of the PI3-K/Akt and Notch/Wnt signaling pathways. Growth Factors.

[CR21] Hovatta O, Mikkola M, Gertow K, Stromberg AM, Inzunza J, Hreinsson J, Rozell B, Blennow E, Andang M, Ahrlund-Richter L (2003). A culture system using human foreskin fibroblasts as feeder cells allows production of human embryonic stem cells. Hum Reprod.

[CR22] Jorissen RN, Walker F, Pouliot N, Garrett TP, Ward CW, Burgess AW (2003). Epidermal growth factor receptor: mechanisms of activation and signalling. Exp Cell Res.

[CR23] Kueh J, Richards M, Ng SW, Chan WK, Bongso A (2006). The search for factors in human feeders that support the derivation and propagation of human embryonic stem cells: preliminary studies using transcriptome profiling by serial analysis of gene expression. Fertil Steril.

[CR25] Li J, Wang G, Wang C, Zhao Y, Zhang H, Tan Z, Song Z, Ding M, Deng H (2007). MEK/ERK signaling contributes to the maintenance of human embryonic stem cell self-renewal. Differentiation.

[CR24] Li Y, Powell S, Brunette E, Lebkowski J, Mandalam R (2005). Expansion of human embryonic stem cells in defined serum-free medium devoid of animal-derived products. Biotechnol Bioeng.

[CR26] Ludwig TE, Levenstein ME, Jones JM, Berggren WT, Mitchen ER, Frane JL, Crandall LJ, Daigh CA, Conard KR, Piekarczyk MS (2006). Derivation of human embryonic stem cells in defined conditions. Nat Biotechnol.

[CR27] Maitra A, Arking DE, Shivapurkar N, Ikeda M, Stastny V, Kassauei K, Sui G, Cutler DJ, Liu Y, Brimble SN (2005). Genomic alterations in cultured human embryonic stem cells. Nat Genet.

[CR28] Martin MJ, Muotri A, Gage F, Varki A (2005). Human embryonic stem cells express an immunogenic nonhuman sialic acid. Nat Med.

[CR29] McElroy SL, Reijo Pera RA (2008). Culturing human embryonic stem cells in feeder-free conditions. CSH Protoc.

[CR30] Mitsui K, Tokuzawa Y, Itoh H, Segawa K, Murakami M, Takahashi K, Maruyama M, Maeda M, Yamanaka S (2003). The homeoprotein Nanog is required for maintenance of pluripotency in mouse epiblast and ES cells. Cell.

[CR31] Park JH, Kim SJ, Oh EJ, Moon SY, Roh SI, Kim CG, Yoon HS (2003). Establishment and maintenance of human embryonic stem cells on STO, a permanently growing cell line. Biol Reprod.

[CR32] Park SP, Lee YJ, Lee KS, Ah Shin H, Cho HY, Chung KS, Kim EY, Lim JH (2004). Establishment of human embryonic stem cell lines from frozen-thawed blastocysts using STO cell feeder layers. Hum Reprod.

[CR33] Prenzel N, Zwick E, Leserer M, Ullrich A (2000). Tyrosine kinase signalling in breast cancer. Epidermal growth factor receptor: convergence point for signal integration and diversification. Breast Cancer Res.

[CR34] Prowse AB, McQuade LR, Bryant KJ, Van Dyk DD, Tuch BE, Gray PP (2005). A proteome analysis of conditioned media from human neonatal fibroblasts used in the maintenance of human embryonic stem cells. Proteomics.

[CR35] Prowse AB, McQuade LR, Bryant KJ, Marcal H, Gray PP (2007). Identification of potential pluripotency determinants for human embryonic stem cells following proteomic analysis of human and mouse fibroblast conditioned media. J Proteome Res.

[CR36] Reinartz J, Bechtel MJ, Kramer MD (1996). Tumor necrosis factor-alpha-induced apoptosis in a human keratinocyte cell line (HaCaT) is counteracted by transforming growth factor-alpha. Exp Cell Res.

[CR37] Reubinoff BE, Pera MF, Fong CY, Trounson A, Bongso A (2000). Embryonic stem cell lines from human blastocysts: somatic differentiation in vitro. Nat Biotechnol.

[CR38] Richards M, Tan S, Fong CY, Biswas A, Chan WK, Bongso A (2003). Comparative evaluation of various human feeders for prolonged undifferentiated growth of human embryonic stem cells. Stem Cells.

[CR39] Richards M, Fong CY, Tan S, Chan WK, Bongso A (2004). An efficient and safe xeno-free cryopreservation method for the storage of human embryonic stem cells. Stem Cells.

[CR40] Santa-Olalla J, Covarrubias L (1995). Epidermal growth factor (EGF), transforming growth factor-alpha (TGF-alpha), and basic fibroblast growth factor (bFGF) differentially influence neural precursor cells of mouse embryonic mesencephalon. J Neurosci Res.

[CR41] Sawhney RS, Cookson MM, Sharma B, Hauser J, Brattain MG (2004). Autocrine transforming growth factor alpha regulates cell adhesion by multiple signaling via specific phosphorylation sites of p70S6 kinase in colon cancer cells. J Biol Chem.

[CR42] Stacey GN, Cobo F, Nieto A, Talavera P, Healy L, Concha A (2006). The development of “feeder” cells for the preparation of clinical grade hES cell lines: challenges and solutions. J Biotechnol.

[CR43] Tang XB, Dallaire P, Hoyt DW, Sykes BD, O’Connor-McCourt M, Malcolm BA (1997). Construction of transforming growth factor alpha (TGF-alpha) phage library and identification of high binders of epidermal growth factor receptor (EGFR) by phage display. J Biochem.

[CR44] Thomson JA, Itskovitz-Eldor J, Shapiro SS, Waknitz MA, Swiergiel JJ, Marshall VS, Jones JM (1998). Embryonic stem cell lines derived from human blastocysts. Science.

[CR45] Villa-Diaz LG, Pacut C, Slawny NA, Ding J, O’Shea KS, Smith GD (2009). Analysis of the factors that limit the ability of feeder cells to maintain the undifferentiated state of human embryonic stem cells. Stem Cells Dev.

[CR46] Wang G, Zhang H, Zhao Y, Li J, Cai J, Wang P, Meng S, Feng J, Miao C, Ding M (2005). Noggin and bFGF cooperate to maintain the pluripotency of human embryonic stem cells in the absence of feeder layers. Biochem Biophys Res Commun.

[CR47] Xu C, Inokuma MS, Denham J, Golds K, Kundu P, Gold JD, Carpenter MK (2001). Feeder-free growth of undifferentiated human embryonic stem cells. Nat Biotechnol.

[CR48] Xu C, Jiang J, Sottile V, McWhir J, Lebkowski J, Carpenter MK (2004). Immortalized fibroblast-like cells derived from human embryonic stem cells support undifferentiated cell growth. Stem Cells.

[CR49] Xu C, Rosler E, Jiang J, Lebkowski JS, Gold JD, O’Sullivan C, Delavan-Boorsma K, Mok M, Bronstein A, Carpenter MK (2005). Basic fibroblast growth factor supports undifferentiated human embryonic stem cell growth without conditioned medium. Stem Cells.

[CR50] Zeng X, Chen J, Liu Y, Luo Y, Schulz TC, Robins AJ, Rao MS, Freed WJ (2004). BG01V: a variant human embryonic stem cell line which exhibits rapid growth after passaging and reliable dopaminergic differentiation. Restor Neurol Neurosci.

[CR51] Zheng Y, Lin L, Zheng Z (2008). TGF-alpha induces upregulation and nuclear translocation of Hes1 in glioma cell. Cell Biochem Funct.

