# Supplementary material for: Study of transforming growth factor alpha for the maintenance of human embryonic stem cells
Source: Cell Tissue Res. 2012 Aug 3;350(2):289–303. doi: 10.1007/s00441-012-1476-7 (PMC3480587; doi:10.1007/s00441-012-1476-7)

Supp. Figure 1

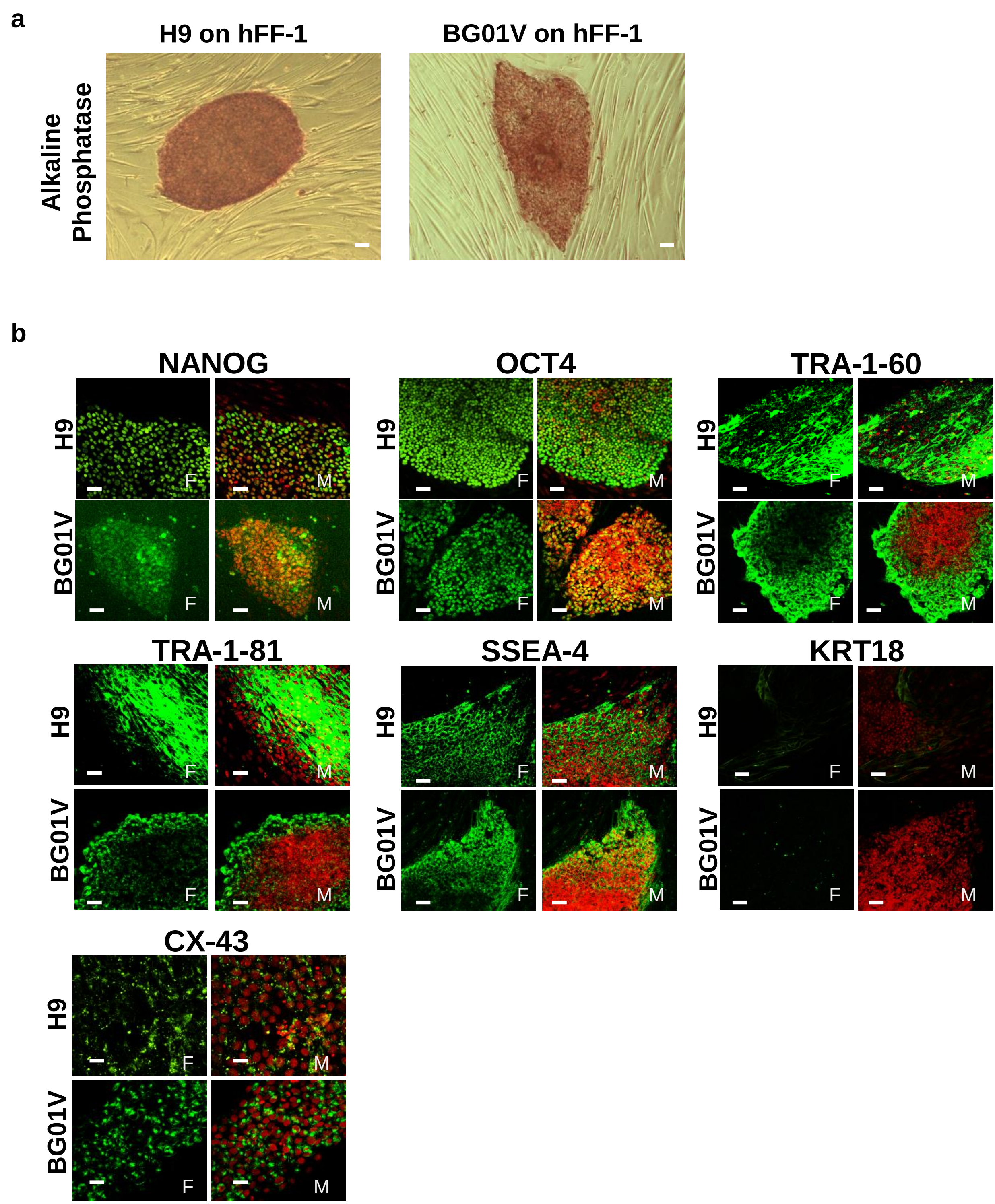

Supp. Figure 1

c

Embryoid Body from H9

Embryoid Body from BG01V

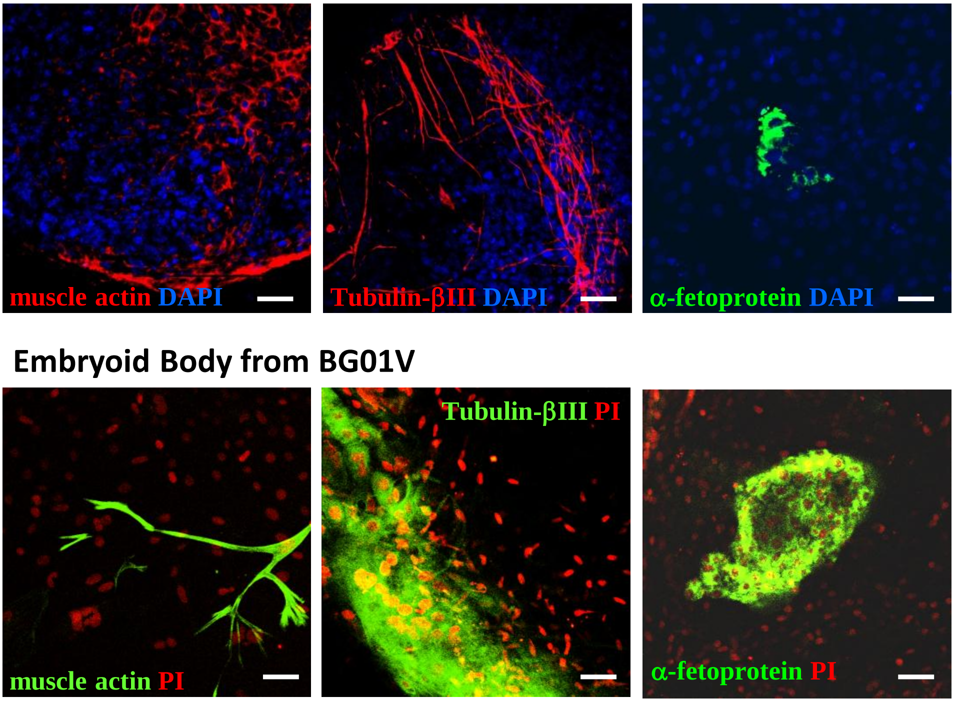

Supp. Figure 2

Embryoid Body from H9 in hFF-1 CM

Embryoid Body from H9 in WI-38 CM

Embryoid Body from H9 in WI-38 CM with TGFα

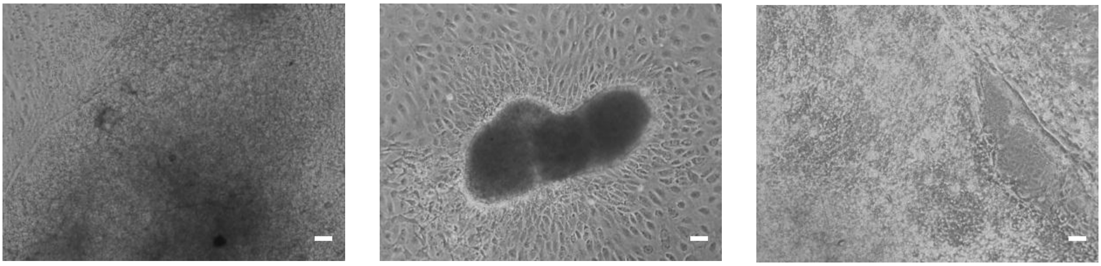

Supplement: Supplementary file 1 — (PDF 698 kb) [file 441_2012_1476_MOESM1_ESM.pdf]
