# Supplementary material for: Study of transforming growth factor alpha for the maintenance of human embryonic stem cells
Source: Cell Tissue Res. 2012 Aug 3;350(2):289–303. doi: 10.1007/s00441-012-1476-7 (PMC3480587; doi:10.1007/s00441-012-1476-7)
Supplement: Supplementary file 2 — (DOC 610 kb) [file 441_2012_1476_MOESM2_ESM.doc]

| **Table S1: Highly expressed genes in hFF-1 cells from microarray analysis (>2 fold)** | | | |
| --- | --- | --- | --- |
| **Fold change**  **([hFF-1] vs [WI-38])** | **Unigene**  **(Avadis)** | **Gene Symbol** | **Gene Title** |
| 334.3311 | Hs.365706 | MGP | matrix Gla protein |
| 158.63544 | Hs.409602 | SULF1 | sulfatase 1 |
| 157.95769 | Hs.76392 | ALDH1A1 | aldehyde dehydrogenase 1 family, member A1 |
| 154.95549 | Hs.512842 | MFAP5 | microfibrillar associated protein 5 |
| 135.93803 | Hs.512842 | MFAP5 | microfibrillar associated protein 5 |
| 134.80551 | Hs.282376 | LOC100133662 /// RPS4Y1 | ribosomal protein S4, Y-linked 1 /// hypothetical protein LOC100133662 |
| 105.24379 | Hs.99120 | DDX3Y | DEAD (Asp-Glu-Ala-Asp) box polypeptide 3, Y-linked |
| 96.6795 | Hs.520293 | SIM1 | single-minded homolog 1 (Drosophila) |
| 94.03682 | Hs.461178 | EIF1AY | eukaryotic translation initiation factor 1A, Y-linked |
| 82.16829 | Hs.442578 | LHX9 | LIM homeobox 9 |
| 71.81318 | Hs.409602 | SULF1 | sulfatase 1 |
| 61.319393 | Hs.512842 | MFAP5 | microfibrillar associated protein 5 |
| 60.779213 | Hs.647962 | ZIC1 | Zic family member 1 (odd-paired homolog, Drosophila) |
| 58.816383 | Hs.128193 | MKX | mohawk homeobox |
| 53.798145 | Hs.521651 | STMN2 | stathmin-like 2 |
| 52.57572 | Hs.439040 | RPESP | RPE-spondin |
| 51.01173 | Hs.310512 | CCRL1 | chemokine (C-C motif) receptor-like 1 |
| 48.183727 | Hs.521651 | STMN2 | stathmin-like 2 |
| 39.764492 | Hs.592166 | HOXA10 | homeobox A10 |
| 38.62677 | Hs.128193 | MKX | mohawk homeobox |
| 38.529915 | Hs.411501 | KRT7 | keratin 7 |
| 37.420986 | Hs.409602 | SULF1 | sulfatase 1 |
| 36.800064 | Hs.40479 | PPAPDC1A | phosphatidic acid phosphatase type 2 domain containing 1A |
| 30.857225 | Hs.659350 | HOXA9 | homeobox A9 |
| 30.333431 | Hs.21365 | NAP1L3 | nucleosome assembly protein 1-like 3 |
| 29.557547 | Hs.170009 | TGFA | transforming growth factor, alpha |
| 28.757706 | Hs.334370 | BEX1 | brain expressed, X-linked 1 |
| 28.738094 | Hs.335163 | LIMCH1 | LIM and calponin homology domains 1 |
| 28.636877 | Hs.592172 | HOXA13 | homeobox A13 |
| 28.26967 | Hs.563491 | EPDR1 | ependymin related protein 1 (zebrafish) |
| 25.578382 | Hs.237396 | hCG_1815491 | hCG1815491 |
| 25.530848 | Hs.584776 | MAB21L1 | mab-21-like 1 (C. elegans) |
| 25.129755 | Hs.196384 | PTGS2 | prostaglandin-endoperoxide synthase 2 (prostaglandin G/H synthase and cyclooxygenase) |
| 24.583134 | Hs.334511 | GPR128 | G protein-coupled receptor 128 |
| 23.91479 | Hs.583755 |  | CDNA FLJ31059 fis, clone HSYRA2000832 |
| 23.479294 | Hs.654631 | ODZ2 | odz, odd Oz/ten-m homolog 2 (Drosophila) |
| 23.306181 | Hs.647061 | ELN | elastin (supravalvular aortic stenosis, Williams-Beuren syndrome) |
| 22.52166 | Hs.335163 | LIMCH1 | LIM and calponin homology domains 1 |
| 22.149952 | Hs.519880 | TFAP2A | transcription factor AP-2 alpha (activating enhancer binding protein 2 alpha) |
| 19.605871 | Hs.439040 | RPESP | RPE-spondin |
| 19.579372 | Hs.647643 | DNAJC6 | DnaJ (Hsp40) homolog, subfamily C, member 6 |
| 19.507072 | Hs.654695 | SPOCK1 | sparc/osteonectin, cwcv and kazal-like domains proteoglycan (testican) 1 |
| 18.93951 | Hs.655309 | LOC100130216 /// USP9Y | ubiquitin specific peptidase 9, Y-linked (fat facets-like, Drosophila) /// hypothetical protein LOC100130216 |
| 18.910149 | Hs.461178 | EIF1AY | eukaryotic translation initiation factor 1A, Y-linked |
| 18.656288 | Hs.288467 | LRRC15 | leucine rich repeat containing 15 |
| 18.519604 | Hs.549040 | HOXC4 /// HOXC6 | homeobox C4 /// homeobox C6 |
| 18.252516 | Hs.335163 | LIMCH1 | LIM and calponin homology domains 1 |
| 18.199636 | Hs.417962 | DUSP4 | dual specificity phosphatase 4 |
| 18.187393 | Hs.164021 | CXCL6 | chemokine (C-X-C motif) ligand 6 (granulocyte chemotactic protein 2) |
| 17.658806 | Hs.19193 | LOC400043 | hypothetical gene supported by BC009385 |
| 17.381275 | Hs.523446 | COL11A1 | collagen, type XI, alpha 1 |
| 17.2457 | Hs.377070 | GRIA3 | glutamate receptor, ionotrophic, AMPA 3 |
| 17.007431 | Hs.119689 | CGA | glycoprotein hormones, alpha polypeptide |
| 16.16804 | Hs.42146 | PAX3 | paired box 3 |
| 16.081951 | Hs.658000 | MYO1D | myosin ID |
| 15.98651 | Hs.237396 | hCG_1815491 | hCG1815491 |
| 15.983314 | Hs.594351 |  | CDNA FLJ31066 fis, clone HSYRA2001153 |
| 15.908242 | Hs.658118 | KRT81 | keratin 81 |
| 15.8724785 | Hs.520293 | SIM1 | single-minded homolog 1 (Drosophila) |
| 15.829689 | Hs.65029 | GAS1 | growth arrest-specific 1 |
| 15.637956 | Hs.594351 |  | CDNA FLJ31066 fis, clone HSYRA2001153 |
| 15.163532 | Hs.339831 | PENK | proenkephalin |
| 14.496686 | Hs.117136 | CCDC85A | coiled-coil domain containing 85A |
| 14.281209 | Hs.162016 | SULF2 | sulfatase 2 |
| 14.2737055 | Hs.522818 | L1CAM | L1 cell adhesion molecule |
| 14.217187 | Hs.369422 | MXRA5 | matrix-remodelling associated 5 |
| 14.189735 | Hs.116092 | EPHB1 | EPH receptor B1 |
| 13.976849 | Hs.190495 | GPNMB | glycoprotein (transmembrane) nmb |
| 13.552809 | Hs.516769 | EFHD1 | EF-hand domain family, member D1 |
| 13.543414 | Hs.522863 | CYorf15A | chromosome Y open reading frame 15A |
| 13.372967 | Hs.532815 | EMILIN2 | elastin microfibril interfacer 2 |
| 12.944538 | Hs.593942 | MFAP3L | microfibrillar-associated protein 3-like |
| 12.932665 | Hs.522891 | CXCL12 | chemokine (C-X-C motif) ligand 12 (stromal cell-derived factor 1) |
| 12.51578 | Hs.444459 | C9orf135 | chromosome 9 open reading frame 135 |
| 12.338943 | Hs.298851 | MLSTD1 | Male sterility domain containing 1 |
| 11.863961 | Hs.593645 | MEGF6 | multiple EGF-like-domains 6 |
| 11.532662 | Hs.185055 | MALL | mal, T-cell differentiation protein-like |
| 11.457122 |  | FLJ41747 | hypothetical gene supported by AK123741 |
| 11.271688 | Hs.591712 | ASB5 | ankyrin repeat and SOCS box-containing 5 |
| 11.269638 | Hs.494312 | NTRK2 | neurotrophic tyrosine kinase, receptor, type 2 |
| 11.14827 | Hs.464422 | COLEC12 | collectin sub-family member 12 |
| 11.124723 | Hs.248160 | SSTR1 | somatostatin receptor 1 |
| 11.115057 | Hs.522845 | LOC100130829 /// ZFY | zinc finger protein, Y-linked /// hypothetical protein LOC100130829 |
| 10.79749 | Hs.298851 | MLSTD1 | male sterility domain containing 1 |
| 10.7294 | Hs.194695 | DIRAS3 | DIRAS family, GTP-binding RAS-like 3 |
| 10.68509 | Hs.414467 |  | Transcribed locus |
| 10.621643 | Hs.659350 | HOXA9 | homeobox A9 |
| 10.562646 | Hs.80358 | JARID1D | jumonji, AT rich interactive domain 1D |
| 10.168877 | Hs.522891 | CXCL12 | chemokine (C-X-C motif) ligand 12 (stromal cell-derived factor 1) |
| 10.0659 | Hs.655388 | ADAM12 | ADAM metallopeptidase domain 12 (meltrin alpha) |
| 10.03794 | Hs.368431 | RUNX1T1 | runt-related transcription factor 1; translocated to, 1 (cyclin D-related) |
| 10.016063 | Hs.499205 | IRX3 | iroquois homeobox 3 |
| 9.412358 | Hs.153521 | KCNC4 | potassium voltage-gated channel, Shaw-related subfamily, member 4 |
| 9.39177 | Hs.654415 | PSG5 | pregnancy specific beta-1-glycoprotein 5 |
| 9.205661 | Hs.296942 | KRT34 | keratin 34 |
| 9.062018 | Hs.164226 | THBS1 | Thrombospondin 1 |
| 9.041989 | Hs.380048 | LOC285382 | hypothetical gene supported by AK091454 |
| 9.032886 | Hs.523446 | COL11A1 | collagen, type XI, alpha 1 |
| 8.919963 | Hs.505 | ISL1 | ISL LIM homeobox 1 |
| 8.66244 | Hs.655309 | LOC100130216 /// USP9Y | ubiquitin specific peptidase 9, Y-linked (fat facets-like, Drosophila) /// hypothetical protein LOC100130216 |
| 8.579559 | Hs.477114 | PHLDB2 | pleckstrin homology-like domain, family B, member 2 |
| 8.512665 | Hs.138760 |  | CDNA FLJ10145 fis, clone HEMBA1003322 |
| 8.416312 | Hs.371147 | THBS2 | thrombospondin 2 |
| 8.364482 | Hs.709357 | FRMD3 | FERM domain containing 3 |
| 8.357327 | Hs.190495 | GPNMB | glycoprotein (transmembrane) nmb |
| 8.340004 | Hs.491172 | NBEA | neurobeachin |
| 8.294308 | Hs.249171 | HOXA11 | homeobox A11 |
| 8.242553 | Hs.69517 |  | Transcribed locus, strongly similar to NP_059997.2 lymphocyte antigen 6 complex, locus K [Homo sapiens] |
| 8.173992 | Hs.411501 | KRT7 | Keratin 7 |
| 7.8528147 | Hs.470457 | COBLL1 | COBL-like 1 |
| 7.767146 | Hs.61596 |  | Transcribed locus |
| 7.7452636 | Hs.519873 | DSP | desmoplakin |
| 7.557037 | Hs.123070 | HOXD10 | homeobox D10 |
| 7.55428 | Hs.353001 | COL8A2 | collagen, type VIII, alpha 2 |
| 7.5093985 | Hs.435655 | ASPN | asporin |
| 7.48058 | Hs.709357 | FRMD3 | FERM domain containing 3 |
| 7.4712987 | Hs.483444 | CXCL14 | chemokine (C-X-C motif) ligand 14 |
| 7.4185176 | Hs.369448 | FRAS1 | Fraser syndrome 1 |
| 7.4024525 | Hs.210283 | COL5A1 | collagen, type V, alpha 1 |
| 7.321288 | Hs.435458 | SETBP1 | SET binding protein 1 |
| 7.3054457 | Hs.418093 | PTGFRN | prostaglandin F2 receptor negative regulator |
| 7.241564 | Hs.486798 | PPP1R14C | protein phosphatase 1, regulatory (inhibitor) subunit 14C |
| 7.1524787 | Hs.591249 | ERAP2 | endoplasmic reticulum aminopeptidase 2 |
| 7.125676 | Hs.445045 |  | Full length insert cDNA clone ZE01A04 |
| 7.1201296 |  | TTTY15 | testis-specific transcript, Y-linked 15 |
| 7.045669 | Hs.700704 |  | CDNA FLJ35153 fis, clone PLACE6010765 |
| 7.0267215 | Hs.198862 | FBLN2 | fibulin 2 |
| 7.0153236 | Hs.654490 | RARB | retinoic acid receptor, beta |
| 6.806893 | Hs.439463 | AEBP1 | AE binding protein 1 |
| 6.793194 | Hs.654413 | PSG3 | pregnancy specific beta-1-glycoprotein 3 |
| 6.732245 | Hs.547787 |  | MRNA; cDNA DKFZp564B213 (from clone DKFZp564B213) |
| 6.6663055 | Hs.384598 | SERPING1 | serpin peptidase inhibitor, clade G (C1 inhibitor), member 1, (angioedema, hereditary) |
| 6.661731 | Hs.262960 | TRPC4 | transient receptor potential cation channel, subfamily C, member 4 |
| 6.6455483 | Hs.590914 | LOC150763 | hypothetical protein LOC150763 |
| 6.6297626 | Hs.529408 | BACE2 | beta-site APP-cleaving enzyme 2 |
| 6.6246347 | Hs.1239 | ANPEP | alanyl (membrane) aminopeptidase (aminopeptidase N, aminopeptidase M, microsomal aminopeptidase, CD13, p150) |
| 6.6001134 | Hs.174312 | TLR4 | toll-like receptor 4 |
| 6.5987334 | Hs.520525 | FNDC1 | fibronectin type III domain containing 1 |
| 6.538454 | Hs.262960 | TRPC4 | transient receptor potential cation channel, subfamily C, member 4 |
| 6.5088882 | Hs.591347 | TPD52L1 | tumor protein D52-like 1 |
| 6.5027857 | Hs.21929 | LYPD6 | LY6/PLAUR domain containing 6 |
| 6.463624 | Hs.591347 | TPD52L1 | tumor protein D52-like 1 |
| 6.3781295 | Hs.459153 | BNC1 | basonuclin 1 |
| 6.3399067 | Hs.105932 | SHOX | short stature homeobox |
| 6.3172784 | Hs.708196 | TWIST2 | twist homolog 2 (Drosophila) |
| 6.3082066 | Hs.513022 | ISLR | immunoglobulin superfamily containing leucine-rich repeat |
| 6.292807 | Hs.522863 | CYorf15A | chromosome Y open reading frame 15A |
| 6.2555647 | Hs.656887 | CPXM2 | carboxypeptidase X (M14 family), member 2 |
| 6.238411 | Hs.654413 | PSG3 | pregnancy specific beta-1-glycoprotein 3 |
| 6.1529174 | Hs.24601 | FBLN1 /// LOC100133843 | fibulin 1 /// similar to Fibulin 1 |
| 6.1279626 | Hs.303649 | CCL2 | chemokine (C-C motif) ligand 2 |
| 6.09959 | Hs.585457 | UNC5B | unc-5 homolog B (C. elegans) |
| 6.043723 | Hs.42419 |  | Transcribed locus |
| 6.002719 | Hs.656754 | GPR133 | G protein-coupled receptor 133 |
| 5.9935746 | Hs.25924 | AJAP1 | adherens junctions associated protein 1 |
| 5.934579 | Hs.414362 | CYB5R2 | cytochrome b5 reductase 2 |
| 5.916946 | Hs.655976 | LOC100131914 | hypothetical protein LOC100131914 |
| 5.900631 | Hs.124638 | C20orf39 | chromosome 20 open reading frame 39 |
| 5.8589263 | Hs.210283 | COL5A1 | collagen, type V, alpha 1 |
| 5.850379 | Hs.164226 | THBS1 | thrombospondin 1 |
| 5.844021 | Hs.200841 | LAMA2 | laminin, alpha 2 (merosin, congenital muscular dystrophy) |
| 5.835285 | Hs.473152 | TFAP2C | transcription factor AP-2 gamma (activating enhancer binding protein 2 gamma) |
| 5.816184 | Hs.675919 | KCNQ5 | potassium voltage-gated channel, KQT-like subfamily, member 5 |
| 5.8141584 | Hs.662980 |  | Transcribed locus |
| 5.8121967 | Hs.439199 | NLGN4Y | neuroligin 4, Y-linked |
| 5.7859826 | Hs.170019 | RUNX3 | runt-related transcription factor 3 |
| 5.733521 | Hs.310458 | TSPAN2 | tetraspanin 2 |
| 5.732126 | Hs.190977 | ENPP2 | ectonucleotide pyrophosphatase/phosphodiesterase 2 (autotaxin) |
| 5.673266 | Hs.380362 |  | Homo sapiens, clone IMAGE:4400004, mRNA |
| 5.6446376 |  | TRBC1 | T cell receptor beta constant 1 |
| 5.63825 | Hs.131819 | SUSD2 | sushi domain containing 2 |
| 5.6316304 | Hs.310421 | APBB1IP | amyloid beta (A4) precursor protein-binding, family B, member 1 interacting protein |
| 5.621498 |  | CYorf14 | chromosome Y open reading frame 14 |
| 5.6053147 | Hs.567973 | SAMD5 | sterile alpha motif domain containing 5 |
| 5.5641985 | Hs.284217 | TSHZ1 | teashirt zinc finger homeobox 1 |
| 5.5482454 | Hs.533566 | H19 | H19, imprinted maternally expressed transcript |
| 5.4951687 | Hs.643588 | PITX2 | paired-like homeodomain 2 |
| 5.4879937 | Hs.591838 | PNMA2 | paraneoplastic antigen MA2 |
| 5.4668283 | Hs.91791 | TMEM16C | transmembrane protein 16C |
| 5.463205 | Hs.494557 | ZNF367 | zinc finger protein 367 |
| 5.38549 | Hs.406013 | KRT18 | keratin 18 |
| 5.3463182 | Hs.642794 |  | Transcribed locus |
| 5.3134956 | Hs.370666 | FOXO1 | forkhead box O1 |
| 5.284578 | Hs.435479 | PPM1H | protein phosphatase 1H (PP2C domain containing) |
| 5.2471504 | Hs.201623 | NHS | Nance-Horan syndrome (congenital cataracts and dental anomalies) |
| 5.214748 | Hs.2820 | OXTR | oxytocin receptor |
| 5.2103853 | Hs.709897 | TBX18 | T-box 18 |
| 5.148899 | Hs.435730 | IRX5 | iroquois homeobox 5 |
| 5.1325502 | Hs.483444 | CXCL14 | chemokine (C-X-C motif) ligand 14 |
| 5.0699 | Hs.407639 | CYP27C1 | cytochrome P450, family 27, subfamily C, polypeptide 1 |
| 5.066915 | Hs.284217 | TSHZ1 | teashirt zinc finger homeobox 1 |
| 5.035747 | Hs.368433 | TPD52 | tumor protein D52 |
| 5.019809 | Hs.138760 |  | CDNA FLJ10145 fis, clone HEMBA1003322 |
| 4.943033 | Hs.24601 | FBLN1 | fibulin 1 |
| 4.8561044 |  |  |  |
| 4.8517456 | Hs.434961 | ATXN1 | ataxin 1 |
| 4.8135834 | Hs.283398 | KANK4 | KN motif and ankyrin repeat domains 4 |
| 4.8017564 |  | tcag7.1238 | homeo box A11, antisense |
| 4.776683 | Hs.529408 | BACE2 | beta-site APP-cleaving enzyme 2 |
| 4.775612 | Hs.351856 | C10orf136 | chromosome 10 open reading frame 136 |
| 4.7239985 | Hs.594846 |  | Transcribed locus |
| 4.722042 | Hs.535724 | FLJ30375 | Hypothetical gene supported by AK054937 |
| 4.693299 | Hs.379191 | SCD5 | stearoyl-CoA desaturase 5 |
| 4.691568 | Hs.307734 | MME | membrane metallo-endopeptidase |
| 4.6795454 | Hs.202354 | DIO2 | deiodinase, iodothyronine, type II |
| 4.6245313 | Hs.655519 | SYNPO2 | synaptopodin 2 |
| 4.5954533 | Hs.289292 | FOXL2 | forkhead box L2 |
| 4.5520926 | Hs.597993 |  | Transcribed locus |
| 4.5389547 | Hs.154652 | FAM110B | family with sequence similarity 110, member B |
| 4.534858 | Hs.435132 |  | CDNA FLJ10151 fis, clone HEMBA1003402 |
| 4.5254464 | Hs.307734 | MME | membrane metallo-endopeptidase |
| 4.519185 | Hs.444541 | LOC401577 | Hypothetical protein LOC401577 |
| 4.5183396 | Hs.419 | DLX2 | distal-less homeobox 2 |
| 4.4870586 | Hs.164226 | THBS1 | thrombospondin 1 |
| 4.4510493 | Hs.694721 | CD24 | CD24 molecule |
| 4.4047694 | Hs.1584 | COMP | cartilage oligomeric matrix protein |
| 4.398722 | Hs.24601 | FBLN1 | fibulin 1 |
| 4.3944287 | Hs.519880 | TFAP2A | transcription factor AP-2 alpha (activating enhancer binding protein 2 alpha) |
| 4.3786426 | Hs.789 | CXCL1 | chemokine (C-X-C motif) ligand 1 (melanoma growth stimulating activity, alpha) |
| 4.3492613 | Hs.380277 | DAPK1 | death-associated protein kinase 1 |
| 4.3439145 | Hs.170986 | GALNT3 | UDP-N-acetyl-alpha-D-galactosamine:polypeptide N-acetylgalactosaminyltransferase 3 (GalNAc-T3) |
| 4.324456 | Hs.656887 | CPXM2 | carboxypeptidase X (M14 family), member 2 |
| 4.2985425 | Hs.367725 | GATA2 | GATA binding protein 2 |
| 4.250312 | Hs.210283 | COL5A1 | collagen, type V, alpha 1 |
| 4.2324414 | Hs.370549 | BCL11A | B-cell CLL/lymphoma 11A (zinc finger protein) |
| 4.21857 | Hs.708143 | SERTAD4 | SERTA domain containing 4 |
| 4.2172527 | Hs.591127 | RASGRP1 | RAS guanyl releasing protein 1 (calcium and DAG-regulated) |
| 4.21139 | Hs.659104 | RNF150 | ring finger protein 150 |
| 4.195802 | Hs.592166 | HOXA10 | homeobox A10 |
| 4.146431 | Hs.23871 |  | CDNA clone IMAGE:30924414 |
| 4.116656 | Hs.116471 | CDH11 | cadherin 11, type 2, OB-cadherin (osteoblast) |
| 4.0968804 | Hs.655519 | SYNPO2 | synaptopodin 2 |
| 4.0753074 | Hs.21929 | LYPD6 | LY6/PLAUR domain containing 6 |
| 4.0661187 | Hs.99120 | DDX3Y /// LOC100130220 | DEAD (Asp-Glu-Ala-Asp) box polypeptide 3, Y-linked /// hypothetical protein LOC100130220 |
| 4.065173 | Hs.513779 | CRISPLD2 | cysteine-rich secretory protein LCCL domain containing 2 |
| 4.043084 | Hs.591283 | RSRC1 | arginine/serine-rich coiled-coil 1 |
| 4.0263634 | Hs.660998 | BMPER | BMP binding endothelial regulator |
| 4.0108347 | Hs.579108 | ARL17 /// ARL17P1 | ADP-ribosylation factor-like 17 pseudogene 1 /// ADP-ribosylation factor-like 17 |
| 3.9766753 | Hs.262960 | TRPC4 | transient receptor potential cation channel, subfamily C, member 4 |
| 3.9411254 | Hs.237028 | NPR3 | natriuretic peptide receptor C/guanylate cyclase C (atrionatriuretic peptide receptor C) |
| 3.9145734 | Hs.482976 | C5orf30 | chromosome 5 open reading frame 30 |
| 3.910924 | Hs.444213 | TLE4 | transducin-like enhancer of split 4 (E(sp1) homolog, Drosophila) |
| 3.9048076 | Hs.114172 | BHMT2 | betaine-homocysteine methyltransferase 2 |
| 3.9000862 | Hs.632481 | PRELP | proline/arginine-rich end leucine-rich repeat protein |
| 3.873556 | Hs.40910 | CPNE8 | copine VIII |
| 3.8652987 | Hs.470457 | COBLL1 | COBL-like 1 |
| 3.8563795 | Hs.496303 |  | CDNA FLJ30378 fis, clone BRACE2007953 |
| 3.8511946 | Hs.546392 | PLAC8 | placenta-specific 8 |
| 3.8328524 | Hs.654412 | PBX1 | Hypothetical LOC100131938 |
| 3.799248 | Hs.699597 | LY6K | lymphocyte antigen 6 complex, locus K |
| 3.7760503 | Hs.78068 | CPZ | carboxypeptidase Z |
| 3.7712464 | Hs.162016 | SULF2 | sulfatase 2 |
| 3.7623901 | Hs.27373 | LOC400451 | hypothetical gene supported by AK075564; BC060873 |
| 3.7348602 | Hs.118681 | ERBB3 | v-erb-b2 erythroblastic leukemia viral oncogene homolog 3 (avian) |
| 3.7228699 | Hs.519168 | FMOD | fibromodulin |
| 3.7223651 | Hs.241545 | FAM26B | family with sequence similarity 26, member B |
| 3.7058477 | Hs.154652 | FAM110B | family with sequence similarity 110, member B |
| 3.7050786 | Hs.102914 | CUL4B | cullin 4B |
| 3.6524954 | Hs.677491 |  | CDNA clone IMAGE:4151535 |
| 3.6408148 | Hs.1735 | INHBB | inhibin, beta B |
| 3.6319718 | Hs.363558 | GRAMD3 | GRAM domain containing 3 |
| 3.6122396 | Hs.377830 | MBOAT1 | membrane bound O-acyltransferase domain containing 1 |
| 3.5815084 | Hs.270084 | NEDD1 | neural precursor cell expressed, developmentally down-regulated 1 |
| 3.564875 | Hs.708143 | SERTAD4 | SERTA domain containing 4 |
| 3.5580184 | Hs.102914 | CUL4B | cullin 4B |
| 3.5579007 | Hs.418093 | PTGFRN | prostaglandin F2 receptor negative regulator |
| 3.5144663 | Hs.116471 | CDH11 | cadherin 11, type 2, OB-cadherin (osteoblast) |
| 3.4826548 | Hs.208358 | C21orf63 | chromosome 21 open reading frame 63 |
| 3.4796915 | Hs.110637 |  | Transcribed locus, strongly similar to NP_689952.1 homeobox A9 [Homo sapiens] |
| 3.479104 | Hs.164226 | THBS1 | Thrombospondin 1 |
| 3.4717903 | Hs.190977 | ENPP2 | ectonucleotide pyrophosphatase/phosphodiesterase 2 (autotaxin) |
| 3.4622152 | Hs.200841 | LAMA2 | laminin, alpha 2 (merosin, congenital muscular dystrophy) |
| 3.4610453 | Hs.235368 | COL5A3 | collagen, type V, alpha 3 |
| 3.4512002 | Hs.654352 | SLC1A4 | solute carrier family 1 (glutamate/neutral amino acid transporter), member 4 |
| 3.4479616 | Hs.518545 | RNASEL | ribonuclease L (2',5'-oligoisoadenylate synthetase-dependent) |
| 3.4471717 | Hs.370666 | FOXO1 | forkhead box O1 |
| 3.4298491 | Hs.709603 | MAF | v-maf musculoaponeurotic fibrosarcoma oncogene homolog (avian) |
| 3.4265532 | Hs.368808 | EHD3 | EH-domain containing 3 |
| 3.4214876 | Hs.43047 |  | CDNA FLJ13585 fis, clone PLACE1009150 |
| 3.4047797 |  | tcag7.1238 | homeo box A11, antisense |
| 3.3816087 | Hs.13349 | NFASC | neurofascin homolog (chicken) |
| 3.3788788 | Hs.27373 | LOC400451 | hypothetical gene supported by AK075564; BC060873 |
| 3.37612 | Hs.658823 | HOXC9 | homeobox C9 |
| 3.3542063 | Hs.268874 | FAM20A | family with sequence similarity 20, member A |
| 3.3453717 | Hs.282089 | IRX2 | iroquois homeobox 2 |
| 3.310731 | Hs.519909 | MARCKS | myristoylated alanine-rich protein kinase C substrate |
| 3.3042095 | Hs.2561 | NGF | nerve growth factor (beta polypeptide) |
| 3.2950697 | Hs.443625 | COL3A1 | collagen, type III, alpha 1 (Ehlers-Danlos syndrome type IV, autosomal dominant) |
| 3.2873838 | Hs.241545 | FAM26B | family with sequence similarity 26, member B |
| 3.262161 | Hs.102914 | CUL4B | cullin 4B |
| 3.2535386 | Hs.177841 | BHLHB3 | basic helix-loop-helix domain containing, class B, 3 |
| 3.2418647 | Hs.464829 | CDH2 | cadherin 2, type 1, N-cadherin (neuronal) |
| 3.2107651 | Hs.55977 | MGC16291 | hypothetical protein MGC16291 |
| 3.1998045 | Hs.712560 | DAAM2 | dishevelled associated activator of morphogenesis 2 |
| 3.194693 | Hs.497806 | MARK1 | MAP/microtubule affinity-regulating kinase 1 |
| 3.1835032 | Hs.388918 | RECK | reversion-inducing-cysteine-rich protein with kazal motifs |
| 3.1802657 | Hs.421136 | HOXD11 | homeobox D11 |
| 3.1553426 | Hs.528366 | SYPL2 | synaptophysin-like 2 |
| 3.1518981 | Hs.441039 | LOC401233 | similar to HIV TAT specific factor 1; cofactor required for Tat activation of HIV-1 transcription |
| 3.1382527 | Hs.56045 | STAC | SH3 and cysteine rich domain |
| 3.1360455 | Hs.170019 | RUNX3 | runt-related transcription factor 3 |
| 3.1334727 | Hs.656339 | RHOJ | ras homolog gene family, member J |
| 3.1173618 | Hs.355455 | ANKRD57 | ankyrin repeat domain 57 |
| 3.115377 | Hs.502266 | C11orf41 | chromosome 11 open reading frame 41 |
| 3.1015592 | Hs.202521 | C8orf79 | chromosome 8 open reading frame 79 |
| 3.1008909 | Hs.88156 |  | Transcribed locus |
| 3.089685 | Hs.657664 |  | Transcribed locus |
| 3.0766485 | Hs.356624 | NID1 | nidogen 1 |
| 3.0695171 | Hs.25924 | AJAP1 | adherens junctions associated protein 1 |
| 3.0672266 | Hs.436416 | ITGA11 | integrin, alpha 11 |
| 3.0466368 | Hs.132966 | MET | met proto-oncogene (hepatocyte growth factor receptor) |
| 3.045918 | Hs.654352 | SLC1A4 | solute carrier family 1 (glutamate/neutral amino acid transporter), member 4 |
| 3.039754 | Hs.655150 | KLHL23 | kelch-like 23 (Drosophila) |
| 3.0298164 | Hs.656805 | ALPK2 | alpha-kinase 2 |
| 3.0271046 | Hs.591318 | BOC | Boc homolog (mouse) |
| 3.0252128 | Hs.175437 | EPB41 | erythrocyte membrane protein band 4.1 (elliptocytosis 1, RH-linked) |
| 3.0120344 | Hs.25155 | NET1 | neuroepithelial cell transforming gene 1 |
| 3.0117786 | Hs.30579 | LOC646762 | hypothetical protein LOC646762 |
| 2.999343 | Hs.155085 |  | CDNA FLJ43660 fis, clone SYNOV4004823 |
| 2.988538 | Hs.176376 |  | Transcribed locus |
| 2.9871223 | Hs.521568 | GCNT1 | glucosaminyl (N-acetyl) transferase 1, core 2 (beta-1,6-N-acetylglucosaminyltransferase) |
| 2.9740422 | Hs.202010 | PLCL2 | phospholipase C-like 2 |
| 2.965598 | Hs.221873 | KCTD15 | potassium channel tetramerisation domain containing 15 |
| 2.9456599 | Hs.283085 | PCDHB6 | protocadherin beta 6 |
| 2.9237866 | Hs.533566 | H19 | H19, imprinted maternally expressed transcript |
| 2.9236784 | Hs.370549 | BCL11A | B-cell CLL/lymphoma 11A (zinc finger protein) |
| 2.8854785 | Hs.593872 |  | Transcribed locus |
| 2.8823235 | Hs.660744 |  | Transcribed locus, strongly similar to NP_694798.1 hypothetical protein LOC230259 isoform 1 [Mus musculus] |
| 2.8794544 | Hs.534619 |  | PNAS-130 |
| 2.8678691 | Hs.612087 | GABRA5 | gamma-aminobutyric acid (GABA) A receptor, alpha 5 |
| 2.8590508 | Hs.492427 | NCALD | neurocalcin delta |
| 2.8363502 | Hs.521568 | GCNT1 | glucosaminyl (N-acetyl) transferase 1, core 2 (beta-1,6-N-acetylglucosaminyltransferase) |
| 2.8356261 | Hs.296049 | MFAP4 | microfibrillar-associated protein 4 |
| 2.8345757 | Hs.482291 | ADAMTS6 | ADAM metallopeptidase with thrombospondin type 1 motif, 6 |
| 2.8015292 | Hs.31961 | SLC8A1 | solute carrier family 8 (sodium/calcium exchanger), member 1 |
| 2.791716 | Hs.268874 | FAM20A | family with sequence similarity 20, member A |
| 2.7770762 | Hs.439643 | SLC16A7 | solute carrier family 16, member 7 (monocarboxylic acid transporter 2) |
| 2.7689877 | Hs.643599 | PAPPA | pregnancy-associated plasma protein A, pappalysin 1 |
| 2.7555363 | Hs.421474 | ABCA5 | ATP-binding cassette, sub-family A (ABC1), member 5 |
| 2.7547157 | Hs.462257 |  | Transcribed locus |
| 2.7545867 | Hs.654790 | SLC24A3 | solute carrier family 24 (sodium/potassium/calcium exchanger), member 3 |
| 2.7515328 | Hs.593907 |  | CDNA FLJ27368 fis, clone UBA03195 |
| 2.7423413 | Hs.361463 | F10 | coagulation factor X |
| 2.7374318 | Hs.497806 | MARK1 | MAP/microtubule affinity-regulating kinase 1 |
| 2.6900847 | Hs.13852 | DNAJB4 | DnaJ (Hsp40) homolog, subfamily B, member 4 |
| 2.6776364 | Hs.102914 | CUL4B | cullin 4B |
| 2.6751647 | Hs.600638 |  | Transcribed locus |
| 2.6680977 | Hs.12256 | MID2 | midline 2 |
| 2.653613 | Hs.58877 | HMCN1 | hemicentin 1 |
| 2.6531954 | Hs.657697 |  | Transcribed locus |
| 2.6453614 | Hs.202354 | DIO2 | deiodinase, iodothyronine, type II |
| 2.6411774 | Hs.118681 | ERBB3 | v-erb-b2 erythroblastic leukemia viral oncogene homolog 3 (avian) |
| 2.6382482 | Hs.138211 | MAPK8 | mitogen-activated protein kinase 8 |
| 2.6351368 | Hs.129895 | TBX3 | T-box 3 (ulnar mammary syndrome) |
| 2.6095998 | Hs.146688 | PTGES | prostaglandin E synthase |
| 2.6056504 | Hs.422585 | TWIST2 | twist homolog 2 (Drosophila) |
| 2.6055148 | Hs.654449 | PGBD3 | piggyBac transposable element derived 3 |
| 2.6045187 | Hs.648369 | HIVEP3 | Human immunodeficiency virus type I enhancer binding protein 3 |
| 2.582367 | Hs.519909 | MARCKS | myristoylated alanine-rich protein kinase C substrate |
| 2.5789564 | Hs.696414 | KIAA0888 | KIAA0888 protein |
| 2.5780544 | Hs.660918 | HOXA7 | homeobox A7 |
| 2.568718 | Hs.443625 | COL3A1 | collagen, type III, alpha 1 (Ehlers-Danlos syndrome type IV, autosomal dominant) |
| 2.5644548 | Hs.14846 | SLC7A1 | solute carrier family 7 (cationic amino acid transporter, y+ system), member 1 |
| 2.560543 | Hs.487869 | ZNRF2 | zinc and ring finger 2 |
| 2.5546691 | Hs.593645 | MEGF6 | multiple EGF-like-domains 6 |
| 2.5528684 | Hs.8562 | RPP25 | ribonuclease P/MRP 25kDa subunit |
| 2.5481906 | Hs.501522 | MGMT | O-6-methylguanine-DNA methyltransferase |
| 2.5383158 | Hs.134060 | FNBP1L | formin binding protein 1-like |
| 2.5372107 | Hs.116471 | CDH11 | cadherin 11, type 2, OB-cadherin (osteoblast) |
| 2.5339854 | Hs.146196 | TBX15 | T-box 15 |
| 2.5137575 | Hs.598298 |  | Transcribed locus |
| 2.5117989 | Hs.208544 | KCNK1 | potassium channel, subfamily K, member 1 |
| 2.5033453 | Hs.655055 | DBNDD2 /// SYS1-DBNDD2 | dysbindin (dystrobrevin binding protein 1) domain containing 2 /// SYS1-DBNDD2 |
| 2.4992986 | Hs.135435 | LOC152742 | hypothetical protein LOC152742 |
| 2.4858184 | Hs.67776 | FAM83H | family with sequence similarity 83, member H |
| 2.4805496 | Hs.22907 | XYLT1 | xylosyltransferase I |
| 2.4670687 | Hs.584760 | GNAZ | guanine nucleotide binding protein (G protein), alpha z polypeptide |
| 2.4258041 | Hs.435458 | SETBP1 | SET binding protein 1 |
| 2.4115515 | Hs.654726 | FLJ21767 /// RASA4 | RAS p21 protein activator 4 /// hypothetical protein FLJ21767 |
| 2.4033868 | Hs.643599 | PAPPA | pregnancy-associated plasma protein A, pappalysin 1 |
| 2.4031165 | Hs.250 | XDH | xanthine dehydrogenase |
| 2.3992794 |  | MARCKS | myristoylated alanine-rich protein kinase C substrate |
| 2.3947613 | Hs.235368 | COL5A3 | collagen, type V, alpha 3 |
| 2.3866603 | Hs.284244 | FGF2 | fibroblast growth factor 2 (basic) |
| 2.3719542 | Hs.523446 | COL11A1 | collagen, type XI, alpha 1 |
| 2.3633316 | Hs.507783 | NHLRC3 | NHL repeat containing 3 |
| 2.3631737 | Hs.77810 | NFATC4 | nuclear factor of activated T-cells, cytoplasmic, calcineurin-dependent 4 |
| 2.3623855 | Hs.632348 | SLC7A8 | solute carrier family 7 (cationic amino acid transporter, y+ system), member 8 |
| 2.35955 | Hs.377070 | GRIA3 | glutamate receptor, ionotrophic, AMPA 3 |
| 2.3578727 | Hs.186579 | FAM111B | family with sequence similarity 111, member B |
| 2.3549788 | Hs.101774 | KIF16B | kinesin family member 16B |
| 2.354898 | Hs.7835 | MRC2 | mannose receptor, C type 2 |
| 2.352737 | Hs.200413 | TMED8 | transmembrane emp24 protein transport domain containing 8 |
| 2.3506854 | Hs.670716 |  | Parathyroid hormone-like peptide mRNA, 3' end |
| 2.3499632 |  | ERCC6 | excision repair cross-complementing rodent repair deficiency, complementation group 6 |
| 2.3195705 | Hs.6314 | LOC652900 /// SEZ6L2 | seizure related 6 homolog (mouse)-like 2 /// similar to seizure related 6 homolog (mouse)-like 2 isoform 1 |
| 2.303084 | Hs.22226 |  | Transcribed locus |
| 2.2845788 | Hs.138211 | MAPK8 | mitogen-activated protein kinase 8 |
| 2.282135 | Hs.513053 | DNAJA4 | DnaJ (Hsp40) homolog, subfamily A, member 4 |
| 2.2736344 | Hs.363558 | GRAMD3 | GRAM domain containing 3 |
| 2.2658384 | Hs.369123 | PLEKHA2 | pleckstrin homology domain containing, family A (phosphoinositide binding specific) member 2 |
| 2.2643967 | Hs.310458 | TSPAN2 | tetraspanin 2 |
| 2.2480547 | Hs.434900 | PDZRN3 | PDZ domain containing RING finger 3 |
| 2.2322204 | Hs.356624 | NID1 | nidogen 1 |
| 2.2227743 | Hs.149252 | CPS1 | carbamoyl-phosphate synthetase 1, mitochondrial |
| 2.2195613 | Hs.6314 | LOC652900 /// SEZ6L2 | seizure related 6 homolog (mouse)-like 2 /// similar to seizure related 6 homolog (mouse)-like 2 isoform 1 |
| 2.2161145 | Hs.506603 | APPL2 | adaptor protein, phosphotyrosine interaction, PH domain and leucine zipper containing 2 |
| 2.2142668 | Hs.132225 | PIK3R1 | phosphoinositide-3-kinase, regulatory subunit 1 (alpha) |
| 2.2078326 | Hs.129895 | TBX3 | T-box 3 (ulnar mammary syndrome) |
| 2.206001 | Hs.654462 | GSTT2 | glutathione S-transferase theta 2 |
| 2.20067 | Hs.657434 | NTNG1 | netrin G1 |
| 2.185121 | Hs.644653 | TCF4 | transcription factor 4 |
| 2.1714795 | Hs.460002 | FLJ11151 | hypothetical protein FLJ11151 |
| 2.1682954 | Hs.24119 | NEK7 | NIMA (never in mitosis gene a)-related kinase 7 |
| 2.1628525 | Hs.659758 | LOC728377 | similar to rho guanine nucleotide exchange factor 5 |
| 2.1595716 | Hs.280387 |  | Transcribed locus |
| 2.1567433 | Hs.368921 | COL16A1 | collagen, type XVI, alpha 1 |
| 2.1538925 | Hs.90572 | PTK7 | PTK7 protein tyrosine kinase 7 |
| 2.1504455 | Hs.459652 | TMEM204 | transmembrane protein 204 |
| 2.149716 | Hs.488877 | POMZP3 | POM (POM121 homolog, rat) and ZP3 fusion |
| 2.149365 | Hs.518403 | PIGZ | phosphatidylinositol glycan anchor biosynthesis, class Z |
| 2.147334 | Hs.439643 | SLC16A7 | solute carrier family 16, member 7 (monocarboxylic acid transporter 2) |
| 2.1409614 | Hs.709357 | FRMD3 | FERM domain containing 3 |
| 2.1384444 | Hs.444668 | TMEM117 | transmembrane protein 117 |
| 2.131579 | Hs.591469 | DDR2 | discoidin domain receptor tyrosine kinase 2 |
| 2.11765 | Hs.533710 | FLRT2 | fibronectin leucine rich transmembrane protein 2 |
| 2.1126661 | Hs.410477 | PLA2R1 | phospholipase A2 receptor 1, 180kDa |
| 2.1043653 | Hs.466662 | NBL1 | neuroblastoma, suppression of tumorigenicity 1 |
| 2.093171 | Hs.657779 | C1orf218 /// DENND1B | chromosome 1 open reading frame 218 /// DENN/MADD domain containing 1B |
| 2.091165 | Hs.643599 | PAPPA | pregnancy-associated plasma protein A, pappalysin 1 |
| 2.0779028 | Hs.489811 | ING3 | inhibitor of growth family, member 3 |
| 2.061906 | Hs.644653 | TCF4 | transcription factor 4 |
| 2.0613034 | Hs.310640 | TIFA | TRAF-interacting protein with forkhead-associated domain |
| 2.0468378 | Hs.711775 | LOC100128501 | hypothetical protein LOC100128501 |
| 2.0450509 | Hs.660541 | P4HA3 | procollagen-proline, 2-oxoglutarate 4-dioxygenase (proline 4-hydroxylase), alpha polypeptide III |
| 2.0442185 | Hs.632788 | FAM19A5 | family with sequence similarity 19 (chemokine (C-C motif)-like), member A5 |
| 2.0426936 | Hs.481720 | MYO10 | myosin X |
| 2.0361812 | Hs.196578 | C10orf85 | chromosome 10 open reading frame 85 |
| 2.0230777 | Hs.71947 |  | MRNA full length insert cDNA clone EUROIMAGE 994183 |
| 2.022561 | Hs.151444 |  | Transcribed locus |
| 2.0211875 | Hs.110364 | PPIC | peptidylprolyl isomerase C (cyclophilin C) |
| 2.0181153 | Hs.705396 | TFEB | transcription factor EB |
| 2.0105407 | Hs.153521 | KCNC4 | potassium voltage-gated channel, Shaw-related subfamily, member 4 |
| 2.008744 | Hs.655608 | EIF4E3 | eukaryotic translation initiation factor 4E family member 3 |
| 2.0022101 | Hs.414489 | KCNS3 | potassium voltage-gated channel, delayed-rectifier, subfamily S, member 3 |

**Table S2: Gene ontology (GO) terms of transcripts with at least two-fold increase in expression in hFF-1 samples**

| **Gene Ontology (GO) Term** | **Count in Selection** |
| --- | --- |
| Extracellular region | 93 |
| Multicellular organismal development | 73 |
| Multicellular organismal process | 73 |
| Developmental process | 73 |
| Transcription regulator activity | 69 |
| Transcription factor activity | 64 |
| Cell adhesion | 47 |
| Biological adhesion | 47 |
| Sequence-specific DNA binding | 46 |
| Proteinaceous extracellular matrix | 36 |
| Extracellular matrix | 36 |
| Extracellular region part | 36 |
| Extracellular matrix structural constituent | 17 |
| Oxygen transport | 6 |
| Oxygen transporter activity | 6 |
